# A Review of Small Molecule Inhibitors and Functional Probes of Human Cathepsin L

**DOI:** 10.3390/molecules25030698

**Published:** 2020-02-06

**Authors:** Dibyendu Dana, Sanjai K. Pathak

**Affiliations:** 1 Chemistry and Biochemistry Department, Queens College of The City University of New York, 65-30 Kissena Blvd, Flushing, NY 11367, USA; 2 Ph.D. Program in Biochemistry, The Graduate Center of the City University of New York (CUNY), 365 5th Ave, New York, NY 10016, USA

**Keywords:** human cathepsin L, cathepsin L probe, cathepsin L inhibitor, activity-based probes, clickable ABP, E-64, CA-074, KDP-1, cathepsin L ABP

## Abstract

Human cathepsin L belongs to the cathepsin family of proteolytic enzymes with primarily an endopeptidase activity. Although its primary functions were originally thought to be only of a housekeeping enzyme that degraded intracellular and endocytosed proteins in lysosome, numerous recent studies suggest that it plays many critical and specific roles in diverse cellular settings. Not surprisingly, the dysregulated function of cathepsin L has manifested itself in several human diseases, making it an attractive target for drug development. Unfortunately, several redundant and isoform-specific functions have recently emerged, adding complexities to the drug discovery process. To address this, a series of chemical biology tools have been developed that helped define cathepsin L biology with exquisite precision in specific cellular contexts. This review elaborates on the recently developed small molecule inhibitors and probes of human cathepsin L, outlining their mechanisms of action, and describing their potential utilities in dissecting unknown function.

## 1. Introduction

Lysosomes play critical roles in human biology receiving, trafficking, processing, and degrading biological molecules from seminal cellular processes, such as endocytosis, phagocytosis, autophagy and secretion. Discovered by the ground-breaking work of de Duve, these single-membrane enclosed cytosolic organelle maintain an acidic (~4.5–5) pH environment, and house close to sixty proteolytic enzymes [1,2]. Among these are the eleven members of the cysteine cathepsin enzymes with a versatile expression and functional profile: Cathepsin L (L1), B, C, F, H, K, O, V (L2), X, S, and W [3]. These enzymes closely mimic the CA1 clan of the papain structure and catalytic cycle and mediate numerous crucial cellular events. For example, they participate in processes involving cell death, protein degradation, post-translational modifications of proteins, extracellular matrix (ECM) remodeling, autophagy, and immune signaling. Given that their functions are aberrantly dysregulated in several human diseases, many are considered prime targets for therapeutic development [4]. Several elegant reviews have recently emerged describing the importance of cysteine cathepsins in both normal physiology and human diseases [5,6,7,8,9,10,11]. The focus of this review is specifically on human cathepsin L, a ubiquitously expressed endopeptidase whose involvement in several human diseases has emerged in recent years. These include liver fibrosis, Type I and II diabetes, cardiac and bone, immune and kidney disorders [12,13,14,15,16,17,18,19,20,21,22,23,24] In addition, its role in a wide variety of highly invasive forms of cancer is also now well established and has been a subject of elegant reviews elsewhere [25,26,27]. Unfortunately, several overlapping and redundant functions of cathepsin L have also emerged [5,28,29,30]. It is therefore critically important that its functional biology in both normal and disease-specific cell types be clearly annotated before significant resources are directed in drug discovery endeavors. In this review, we will briefly outline the biogenesis of cathepsin L, describe its post-translational processing and trafficking, and highlight key structural features required for formation of an active and mature cathepsin L. A brief summary of cathepsin L biology and its role in human diseases is provided next. Finally, a detailed and up-to-date report on existing cathepsin L-targeting small molecule inhibitors and functional probes with their mechanistic details is described.

**Figure 1:**
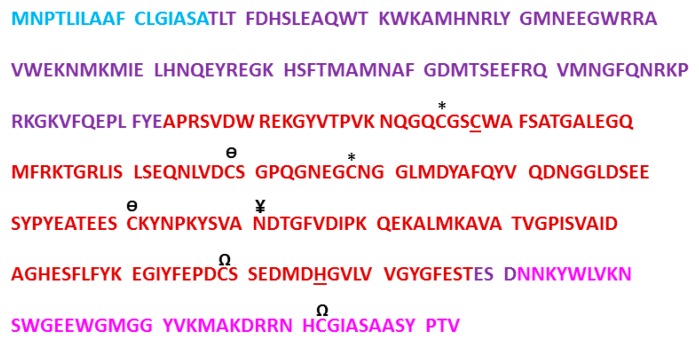
The primary sequence of the full length human inactive human cathepsin L: Blue = 17 Amino acid signal (prepro) peptide, Purple = Propeptides/activation peptides: 96 amino acid (Thr18–Glu113) and 3 amino acid (Glu289–Asp291), Red = Heavy chain peptide, Pink = Light chain peptide; * Disulfide bond pair residues, Cys135–Cys178, ^Ɵ^ Disulfide bond pair Cys169–Cys211, ^Ω^ Disulfide bond pair Cys268–Cys322, ^¥^ The site of N-linked glycosylation. The two key catalytic residues of the active site, Cys25 and His163 (numbering of mature cathepsin L) residing in the heavy chain are underlined.

**Figure 2:**
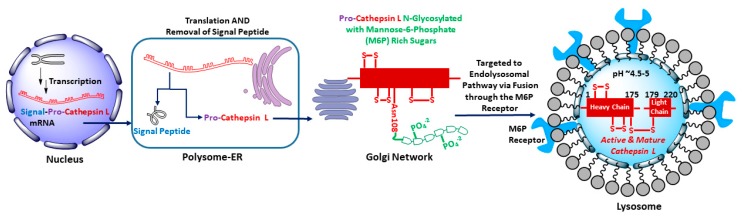
Biogenesis of human cathepsin L. After the full length cathepsin L mRNA is transcribed, it is translated in ribosomes. Following this, the full-length peptide enters the ribosomes-bound endoplasmic reticulum lumen where signal peptide is removed. Pro-cathepsin L then enters the Golgi network where it undergoes N-linked glycosylation at Asn108, followed by mannose phosphorylation and formation of appropriate disulfide linkages. In the last step, modified procathepsin L is shuttled to lysosome by endolysosomal pathways, generating the double chain form of active and mature human cathepsin L.

Human cathepsin L gene, CTHL (Uniprot primary accession number: P07711), located at the 9q21-q22 position of chromosome 9, encodes for a total of 333 amino acid peptide sequence (M.W. = 37,564 kDa) [31]. The coding space includes the regions of a N-terminal signal peptide, two pro-peptides, and two mature peptides comprising of a heavy (H) chain and a light (L) chain (Figure 1). After transcription, the signal peptide is co-translationally removed in polysome and the resulting 41 kDa pro-cathepsin L is translocated to endoplasmic reticulum where it undergoes N-linked glycosylation with mannose rich sugars. The mannose sugars on the pro-cathepsin L are then phosphorylated in *cis* Golgi by UDP-N-acetylglucosamine:N-acetylglucosaminephosphotransferase enzyme [32]. The mannose-6-phosphate (M6P) receptors located on the surface of the *trans* Golgi network recognize the M6P-pro-cathepsin peptide and deliver the pro-cathepsin L peptide to the lysosome via the endolysosomal pathway. The weakly acidic environment of endosome/lysosome releases M6P receptors and the phosphate group from mannose sugars is removed by a lysosomal acid phosphatase [33,34]. Activation to mature cathepsin L form then occurs by removal of propeptides either by autocatalysis [35] or by aspartyl cathepsin D in the acidic environment of lysosome [36]. This leads to the double chain form of mature and active cathepsin L, comprising of H and L domains, connected by disulfide bridges, (Figure 2). It is to be noted here that several isoforms of cathepsin L have also been observed in specific cell types due to alternative splicing of mRNA transcripts and alternative translation [4,37,38,39,40,41].

The propeptides act as an important regulatory on/off switch as well as a folding catalyst in cathepsin activation. Not surprisingly, the nature of propeptides among cysteine cathepsins is highly divergent by both chain lengths and primary sequences. It is thought that this uniqueness is functionally relevant given its ubiquitous presence in most tissues and allows for the selective suppression of enzyme activity (hence unintended autoactivation) during the transport to the endolysosomal compartment. In cathepsin L, two inhibitory propeptides, one containing 96 amino acid (Thr18–Glu113) and the other containing 3 amino acid (Glu289–Asp291) exist. A crystal structure of human procathepsin L revealed that the 96 amino acid inhibitory propeptide chain spans in the opposite directions of substrate binding and forms several high-affinity non-covalent interactions with the surrounding residues in active site [42,43]. Interestingly, this opposite direction binding of inhibitory propeptide segment is evolutionarily conserved in other members of cysteine cathepsins, including in cathepsin B.

The dominant pathway of regulation of activated and mature cathepsin L is by endogenous protein inhibitors, cystatins, that like propeptide compete with the physiological substrates for binding to the enzyme active site (Table 1) [5,44]. Interestingly, protein inhibitory agents of cathepsin L have also been reported in other organisms. For example, Kotsyfakis M. et al. reported the existence of two cathepsin L inhibitory proteins in the carrier of the main vector of Lyme disease-carrying parasite, *Ixodus scapularis*. Named so because of its abilities to specifically inhibit cathepsin L (IC_50_ = 4.68 nM; K_i_ = 95 pM) activity, sialostatin L abrogates the protective proteolytic activity of host cells at the infestation sites, thereby promoting the tick’s survival [45]. In addition, it also possess a potent anti-inflammatory and immunosuppressive activity by inhibiting cytotoxic killer T cells [46].

## 2. Functional Biology of Cathepsin L and Its Role in Human Diseases

Different forms of cathepsin L enzyme have been isolated in distinct cell types and their intracellular and extracellular environment [4]. At specific cellular locations, they seem to play pivotal roles in a wide range of functional activities. In addition to the degradation of cellular proteins by the endolysosomal pathway, cathepsin L also participates in the autophagic degradation pathway; it specifically degrades two autophagosomal markers, LC3-II and GABARAP-II [57]. The specialized podocyte cells in the kidney play a critical role in the retention of various proteins required to be maintained in blood plasma [58]. It was shown that the cytoplasmic variant of active cathepsin L can degrade two key proteins, GTPase dynamin and synaptopodin, required for podocyte’s proper glomerular filtration function [23,59,60]. Consistently, a higher podocyte cathepsin L expression was found in a variety of proteinuric kidney diseases, and its functional inhibition (e.g., by creating cathepsin L resistant dynamin) led to reversing of the symptoms [23,61]. Interestingly, a nuclear isoform of cathepsin L is associated with another unique function in kidney cells; it is involved in the proteolytic processing of Cux1, a cell cycle regulatory transcription factor that promotes cell proliferation and polycystic kidney disease (PKD) [62,63]. Indeed, in mouse model of PKD, a lower level of nuclear cathepsin L was corelated with an increased level of Cux1 protein in cysts [62].

Another type of cells where cathepsin L seems to play a major role in maintaining homeostasis is cardiomyocytes [17,64,65,66,67]. Early investigation by Stypmann et al. on a year old *Ctsl−/−* mice showed that they developed many key traits of dilated cardiomyopathy, such as interstitial myocardial fibrosis, cardiac chamber dilation, and impaired contraction. In addition, the newborn *Ctsl−/−* mice acquired increased number of acidic organelles, although with altered morphology and function. These studies indicate that the inhibition of cathepsin L activity in these cells is detrimental to the proper functioning of cardiac cells. This was further corroborated by an overexpression study of cathepsin L in mice cardiomyocytes that exhibited cardioprotective effect by inhibiting the Akt signaling pathway [68]. The function of secreted extracellular cathepsin L in cardiac remodeling and repair has also been studied extensively since it is known that many proteins of extracellular matrix (ECM) are also the physiological substrate of cathepsin L; these include laminin, collagen type I, IV and XVIII, and fibronectin [69,70,71]. Ischemic heart disease is major risk factor for diabetic patients, unable to maintain their sugar levels efficiently. During cardiac repair, endothelial progenitor cells containing elevated levels of cathepsin L home on ischemic cells, and initiate the process of neovascularization [72,73,74]. In uncontrolled diabetic condition however, cathepsin L function in ECM space during neovascularization is significantly impaired; this hampers the ischemic repair process [15,75].

Controlled release of trypsinogen from pancreatic acinar cells and its subsequent activation in duodenum is a critically important event and must be tightly regulated. Dysregulated release of activated trypsin results in an acute pancreatitis condition. While cathepsin B efficiently cleaves trypsinogen to produce enhanced levels of active trypsin promoting pancreatitis, cathepsin L acts a regulatory brake, and keeps the activation process under control. And it does so by cleaving trypsinogen at a distinct site, 3 amino acids C terminal to cathepsin B, thereby inactivating trypsinogen itself [76]. Consistently, *Ctsl−/−* mice showed increased trypsin activity in pancreas and surprisingly reduced pancreatitis due to diminished inflammation; this effect was attributed due to enhanced apoptosis of acinar cells (vs necrosis). 

In neuro- and immuno-biology, cathepsin L has important documented roles as well [77,78,79,80]. For example, regulated secretory vesicles of the neuroendocrine system, such as chromaffin granules, house many mature function-ready enkephalin opioid peptides. Upon appropriate signaling stimuli, the activated enkephalin neuropeptides are secreted and released imparting their analgesic and immune-cell specific functions. Studies by Yasothornsrikul et al. revealed that cathepsin L is the proteolytic processing enzyme that renders active metenkephalin from proenkephalin [81]. In another studies, the severity of symptoms due to antigen-induced arthritis (AAI) was significantly reduced in *Ctsl−/−* mice due to a weakened T helper cell population in thymus [82].

The expression of cathepsin L is highly dysregulated in several human diseases, including diabetes, AAI, abdominal aortic aneurysm, liver fibrosis, and cancers [13,82,83,84,85]. Its involvement in numerous forms of highly invasive cancers is particularly noteworthy and is covered in great detail in recent reviews [25,26,86,87,88]. Early studies by Gottesman revealed that extracellular cathepsin L levels can increase up to 200-fold in transformed cells [89]. In the ECM environment, they can rapidly degrade several structural components and significantly increase the migratory/invasive potential of these cells, promoting metastasis [86,87]. Not surprisingly, cathepsin L inhibition by small molecule is considered a viable strategy for the development of novel anti-cancer agents. 

## 3. Small Molecule Inhibitors 

**Figure 3:**
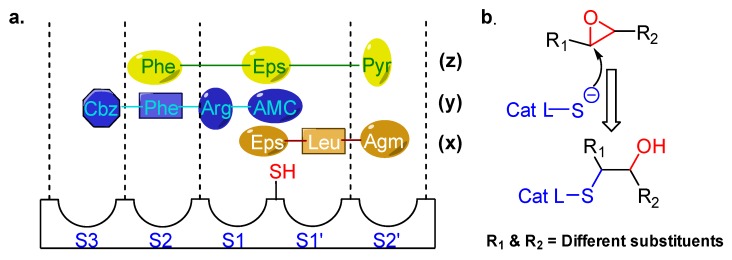
(**a**) Proposed binding mode of (**x**) E-64, (**y**) a putative cathepsin L substrate, and (**z**) CLIK148 to the active site of cathepsin L. (**b**) Inactivation mechanism of cathepsin L by epoxy-containing inhibitors.

**Figure 4:**
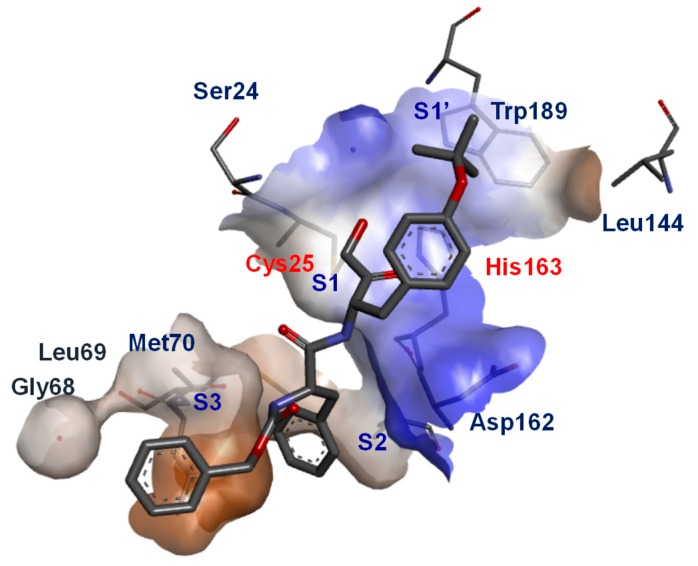
Schematic representation of Z-Phe-Tyr (O-tert-Butyl)-C(O)C(H)O bound within the cathepsin L subsites. The key subsite-forming amino acid residues with their corresponding numbers are shown in blue whereas the catalytic diad forming residues are depicted in red. (The authors used PDB:3OF8 file to construct the figure [96]).

Over the years, several classes of physiological and synthetic inhibitors have been discovered targeting cathepsin L. Herein, the focus is on non-physiological inhibitors that could broadly be classified as reversible and irreversible inhibitors. Reversible and irreversible inhibitor could be distinguished by means of the mechanistic approaches they utilize for enzyme inactivation. Reversible inhibitors ‘generally’ engage with the target protein using non-covalent interaction; however, in certain cases, they form a quasi-covalent bond which eventually disengages from the active site, say, upon dilution. Irreversible inhibitors, on the other hand, permanently modify the protein of interest via the formation of a stable covalent bond. While both reversible and irreversible inhibitors have their pros and cons, it is generally believed that reversible inhibitors are preferred candidates in drug discovery and irreversible inhibitors not so due to their adverse immune responses [90]. There are, however, several examples of successful drugs being used in clinics that work by an irreversible inhibitory mechanism [91]. Irreversible inhibitors are also widely utilized in functional biology (e.g., in the development of activity-based probes). The majority of synthetic cathepsin L inhibitors have recognizable peptide sequences, often derived from its physiological substrates, that bind to the active site of the protein and often contain strategically placed electrophilic warheads that trap the nucleophilic Cys25 residue (Figure 3) for activity. Knowledge of enzyme structure and its associated substrate specificity thus has played an important role in designing selective inhibitors of cathepsins [92,93]. In a seminal study, Choe et al. studied substrate specificity using a highly diversified positional scanning synthetic combinatorial library comprised of 160,000 fluorogenic tetrapeptides; this allowed to differentiate individual enzymes′ binding propensity based on their distinct amino acid preferences [93]. By capitalizing on this strategy, they successfully developed a selective substrate and substrate-based inhibitor of cathepsin K. The other important contribution to our understanding of subsite binding preferences of cathepsin L enzyme stems from several timely crystal structure studies that helped reveal key difference in the structural landscape of enzyme subsites [94,95,96]. In an important study, Shenoy et al. solved the crystal structure of ligand bound cathepsin L and documented the structural compositions of ligand binding sites (Figure 4) [96]. Their analysis of Z-Phe-Tyr (O-*tert*-Butyl)-C(O)C(H)O bound cathepsin L revealed that (a) S1 subsite is relatively wide and unrestricted and composed of Asp162, Ser24, and Cys25, (b) S1′ subsite is guided by Leu144, Trp189, Ala138 and Gly139 where Trp189 associates with Trp193 and Phe143 and forms an aromatic cluster that accommodates the *tert*-butyl group (c) side chains of Leu69 and Met70 help form the S2 subsite that engages in non-polar interactions with the phenyl side chain, and finally, (d) the carboxybenzyl group finds interaction with the Gly68 residue of S3 subsite (Figure 4). However, it turns out that different ligands may find slightly altered binding interactions with the enzyme subsites, depending on their structural features. For example, Shenoy et al. showed that although Z-Phe-Tyr (O-*tert*-Butyl)-DMK binds to the same subsites of cathepsin L as observed for the Z-Phe-Tyr (O-*tert*-Butyl)-C(O)C(H)O ligand and finds some alternative interactions with residues from the active site pocket. This finding has been corroborated by other studies as well that suggest that structural features of ligands can influence the subsite composition of the cathepsin L enzyme [94,95,97,98]. Such information has been duly capitalized to develop different classes of inhibitors that are discussed next. In the following section, we discuss the types of cathepsin L-targeting chemotypes that have been utilized for development of small molecule inhibitors. 

### 3.1. Epoxysuccinates 

Epoxysuccinate inhibitors have historically played a crucial role in deciphering the cysteine protease biology. This class of compounds contains an epoxide ring as an electrophilic warhead that traps the active site catalytic cysteine residue of the protein. E-64 (L-trans-Epoxysuccinyl-leucylamido(4-guanidino)butane) (Entry 1; Table 2), perhaps the most studied commercially available universal cathepsin inhibitor of this class, showed only a marginal selectivity towards cathepsin L compared to cathepsin B and a moderate 24-fold selectivity over cathepsin H, as reported by Barrett et al. [99]. Although they developed several synthetic analogs of E-64 with improved potency, they lacked desirable selectivity toward cathepsin L. They proposed the probable binding orientation of E-64 that follows a non-substrate-like orientation, i.e., it occupies only non-prime sites (Figure 3) at the enzyme pocket.

In another study, Gour-Salin et al. reported a number of epoxysuccinyl amino acid benzyl esters in which they systematically varied the amino acid attached to the epoxide ring. This was intended to investigate its effect in determining selectivity toward cathepsin L or S [100]. Their results surprisingly indicate that the specificity of these analogs did not follow the trend, generally observed for substrate, possibly due to E-64 like binding orientation at the enzyme pocket. Among all synthesized compounds, HO-Eps-Arg-OBzl (Entry 2, Table 2) showed a significant 89-fold selectivity for cathepsin L over cathepsin S.

Notably in a seminal study, Katanuma et al. developed several cathepsin L-selective E-64 analogs (Cathepsin L Inhibitor by Katunuma: CLIK) with the help of computational modeling based on the stereo-structure [101]. Three of the developed CLIK inhibitors were hydrolytically stable and showed highly selective inhibition for hepatic cathepsin L in vivo. Further, they elucidated the inhibition mechanism of this class of compounds based on the crystal structure of papain-CLIK148 (Entry 3, Table 2) complex [102]. This crystal structure revealed that CLIK148, unlike E-64, binds to both prime and non-prime sites of the active site pocket. Notably, the specificity toward cathepsin L was attributed to the existence of phenylalanine residue at the S2 site, and a hydrophobic interaction mediated by N-terminal pyridine ring (Figure 3).

### 3.2. Peptidyldiazomethane and Peptidylchloromethane

The other classes of alkylating agents that initially played an important role in deciphering the mechanistic aspects of cathepsin inhibition were peptidyldiazomethane (Entry 4, Table 2) and peptidylchloromethane (Entry 5, Table 2). Crawford et al. reported highly potent inhibitors from these two chemotypes that spans the active site and shows significant selectivity improvement over other cysteine proteases [103]. However, these classes of inhibitors suffer from stability issues and have found limited utility in in vivo assays.

### 3.3. Peptidylhydroxylamine and Peptidylhydroxamates

**Figure 5:**
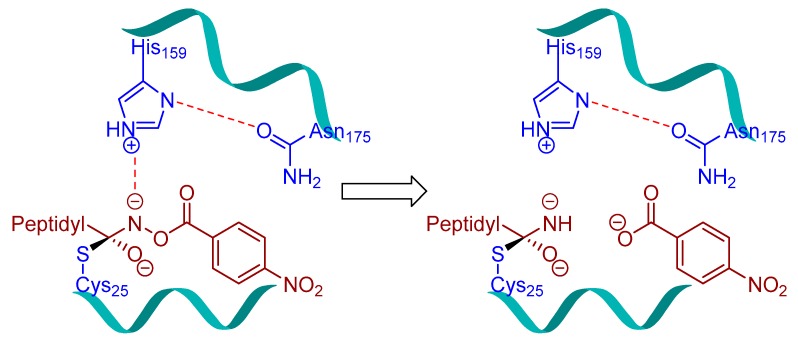
Postulated active-site interactions between papain, a prototypical cysteine protease, and peptidyl hydroxamate inhibitor. Active-site cysteine forms a covalent bond with the carbonyl group of the inhibitor and the negatively charged nitrogen engages in electrostatic interaction with the positively charged histidine residue.

Peptidylhydroxylamines were first introduced as mechanism-based inhibitors of serine and cysteine proteinases [104,105,106,107]. Bromme et al. adapted this scaffold and prepared a library of *N*-peptidyl-*O*-acyl hydroxylamines which exhibited rapid and selective inactivation of several lysosomal cysteine proteinases [108]. This class of inhibitors occupied the active site of the enzyme and irreversibly inactivated cysteine cathepsins as the free enzyme activity was not recovered when the enzyme-inhibitor complex was exposed to exhaustive ultrafiltration (up to an enzyme/free inhibitor ratio of <1:0.05) or chromatography on Sephadex G-10. Among developed inhibitors, Z-Phe-Phe-NHO-MA (Entry 6, Table 2) inhibited cathepsin L with most potency and showed significant selectivity, 58-fold and 436-fold, over cathepsin S and cathepsin B, respectively. Interestingly the stability and efficacy of this class of inhibitors were determined by the nature of substitutions on hydroxylamine oxygen as their electron-withdrawing tendencies showed a positive correlation with inactivation kinetics. Although this class of inhibitors exhibited desirable traits with respect to both potency and selectivity, they suffered majorly from aqueous stability issue; half-lives (t_1/2_) only in the range of 45209 min in aqueous solution. In another study, Bromme et al. developed a series of *N*-peptidyl-*O*-acyl hydroxamates with lysine in P1 position with improved inhibitory profile for cysteine proteases over their serine counterparts [109]. The maximum inhibition was observed by Z-Phe-Lys-NHO-NBz (Entry 8, Table 2), with 7-fold selectivity over cathepsin S, and >100-fold selectivity over cathepsin B. The authors postulated that the active site Cys residue attacks the carbonyl of the hydroxamate and forms a tetrahedral intermediate, whereas the nitrogen of the hydroxamate, which primarily remains deprotonated (pKa < 5), presumably engages in an electrostatic interaction with the active site His159 residue (Figure 5).

In a follow-up study, Bromme et al. synthesized and tested a series of new inhibitors, with the general formula of Z-Phe-Gly-NHO-CO-Aa (Aa: amino acid), against papain class of enzymes [110]. This class of inhibitors covalently modified active-site Cys residue via sulfenamidation, like in their *N*-peptidyl-*O*-acyl hydroxamate counterparts, as shown by the mass spectrometric analysis. These inhibitors further provided the option of varying the leaving group that targets S2′ site; more hydrophobic substituents were preferred at this position as comparison of k_i_/K_i_ values of the inactivation exhibited the following trend for Aa: Gly < Ala < Val < Leu < Phe < 4-NO_2_-Ph. The nitrophenyl analog (Entry 7, Table 2) which incur both hydrophobicity and electron-withdrawing property exerted the maximum potency and selectivity for cathepsin L over other tested cysteine proteases.

### 3.4. Peptidyl Acyloxymethanes/Acyloxymethyl Ketones

**Figure 6:**
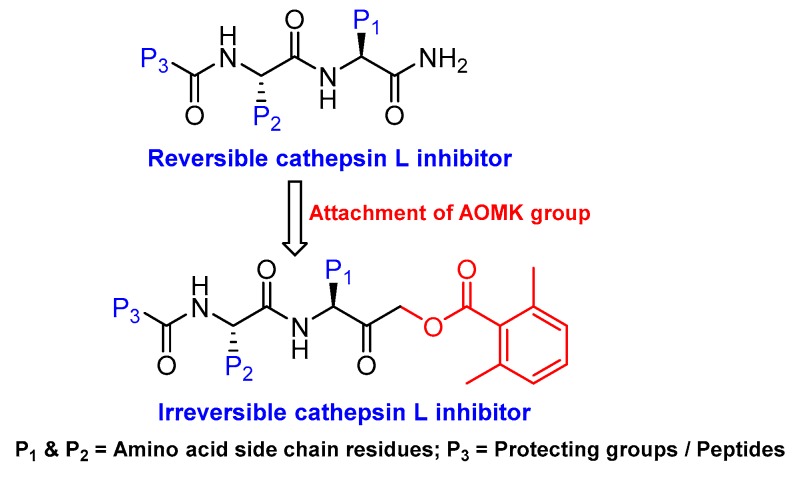
A reversible cathepsin L inhibitor was transformed to an irreversible one by strategically galvanizing an electrophilic warhead.

Another class of peptidyl inhibitors that spans the active site and utilizes the catalytic machinery of cathepsin L for effective attenuation of enzyme activity is peptidyl acyloxymethanes. Krantz et al. developed a library of inhibitory compounds with a general sequence, Z-Phe-X-CH_2_OCO-R; here they systematically varied the amino acid residue at P1 (denoted as X) and P1′ (denoted as R) positions [112]. Among the synthesized compounds, Z-Phe-Cys(SBn)-CH_2_OCO-2,6-(CF_3_)_2_-Ph (Entry 9, Table 2) exhibited almost a diffusion-controlled inactivation kinetics toward cathepsin L, however it showed only marginal selectivity over cathepsin B and cathepsin S. By analyzing the structure-activity relationships of the library, the author elucidated the importance of S1′ site in cathepsin L that could potentially be used to harness selectivity among other cysteine cathepsins. In a separate study, Torkar et al. designed a library of peptides for cathepsin L that spanned the active site and attenuated the enzyme activity via a non-covalent interaction [113]. They initially evaluated the compounds’ activities against cathepsin L and cathepsin B, and compared their hits against cathepsin K and S. The authors discovered five most selective non-covalent, peptidyl inhibitors of cathepsin L, and transformed them into irreversible inhibitors by strategically appending electrophilic warhead (Figure 6)—acyloxymethyl ketone (AOMK) groups (Entry 10, Table 2). However, the attachment of the AOMK group drastically impacted the selectivity profiles of these inhibitors, suggesting the importance of the adjuvant effect of prime site targeting in determining the efficacy and selectivity of this class of compounds. This class of inhibitors found wide-spread utilities in detecting protease activity and were utilized to develop activity-based probe; this will be discussed in the later sections.

### 3.5. Peptidyl Aziridine

**Figure 7:**
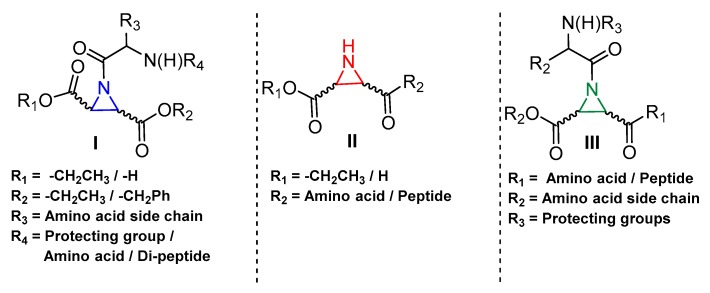
Types of N-substituted and unsubstituted aziridines (color-coded for visual clarity) that bind to the active site of cathepsin L and assume different orientations depending on the favorable interactions incurred by the R-groups.

A very interesting class of inhibitors, the concept of which was derived from E-64, is peptidyl aziridines. Martichonok et al. developed a series of aziridine derivatives of E-64 and tested them against papain and cathepsin L and B [114]. Contrarily to E-64, in which (L)-diastereomer is more potent than (D)-isomer, aziridine analogs exhibited the opposite trend while still inactivating the enzymes. More importantly, the efficacy of this class of inhibitors was strongly pH-dependent and showed maximal inhibitory potency at pH4; this is attributed to the protonated form of aziridine ring that is more susceptible to nucleophilic attack by catalytic Cys residue. Among the developed library, HO-(*D*)-Az-Leu-NH-iAm (Entry 11, Table 2) analog exhibited maximal inhibitory potency towards cathepsin L with moderate selectivity over cathepsin B; the corresponding L isomer, HO-(*L*)-Az-Leu-NH-iAm (Entry 11) was almost 10-fold less activity while maintaining the similar trend in general. Since only protonated form of aziridine ring undergoes nucleophilic attack, presumably it does not involve water molecule-mediated ring-opening like its epoxide counterpart E-64. Although promising, a full potential of this class of compounds could presumably be achieved only below pH 4 when aziridine ring nitrogen gets completely protonated. This class of inhibitors thus lacks practical utility as the catalytic cysteine of cathepsin L starts to lose its activity below pH 4 and the majority of the cell-based assays are performed mostly at a pH higher than 4.0. In a notable study, Schirmeister et al. developed three different classes of inhibitors with aziridine-2,3-dicarboxylic acid (Azi), an electrophilic warhead, installed at different positions of the peptide chain (Figure 7) [115]. They performed a thorough SAR analysis of this type of inhibitors, all off which exhibited time-dependent irreversible inactivation of cathepsin L with no-recovery of enzyme activity, even after extensive dialysis of the enzyme-inhibitor complex. Among type-I inhibitors, N-acylated aziridines with aziridine as C-terminal amino acid, a mixture of diastereomeric peptides with procathepsin B sequence Leu-Gly-Gly (Entry 12, Table 2), exhibited enhanced inhibition toward cathepsin L. This was attributed to an overall unique folding of cathepsin L with a shallowness of the S2 pocket due to the presence of an additional Met161 residue. Type II class of inhibitors (Figure 7) resemble classic aziridine scaffold, N-unsubstituted aziridines with aziridine as N-terminal amino acid, analogously to E-64 where nitrogen of aziridine remained unsubstituted. Among the type II inhibitors tested, EtO-(*R*,*R*)-Azi-Leu-OBzl (Entry 13, Table 2) inactivated cathepsin L with higher second-order rate constant than EtO-(*S*,*S*)-Azi-Leu-OBzl, although the latter showed better selectivity over cathepsin B. 

**Figure 8:**
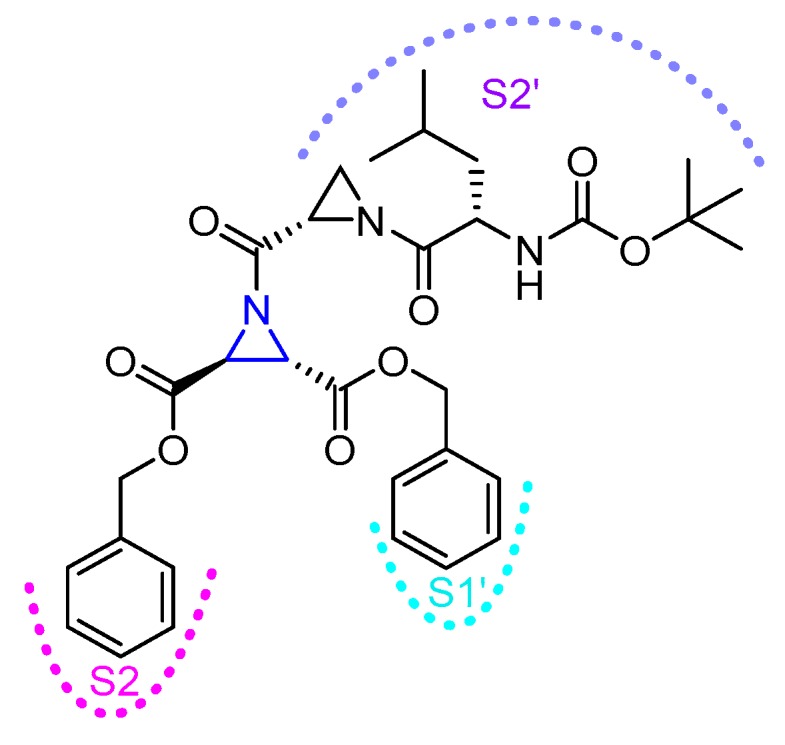
Binding mode of Boc-(*S*)-Leu-(*S*)-Azy-(*S*,*S*)-Azi(OBn)_2_ in cathepsin L active site, as predicted by docking studies (Vicik et al., *ChemMedChem* **2006**, *1*, 1021–1028).

The type III inhibitor class (Figure 7) is comprised of N-acylated bispeptidyl derivatives of aziridine, where aziridine ring rests in the middle of the peptide. BOC-Phe-(*R*,*R*)-(EtO)-Azi-Leu-Pro-OBzl (Entry 14, Table 2) of this series was 5-fold more potent than the (*S*,*S*) analog; however both exhibited only a marginal selectivity over cathepsin B with diminished eudysmic ratio. The authors then superimposed and analyzed the structures of certain epoxide and aziridines and postulated that these inhibitors can assume different orientations in the active site while still binding within the enzyme pockets. This scaffold was further explored by Vicik et al. who extended the previous work and developed a series of compounds in which Boc-(*S*)-Leu-(*S*)-Azy-(*S*,*S*)-Azi(OBn)_2_ (Figure 8), Type I analog, spanned from S2 to S2′ pocket and inactivated cathepsin L with more than 700-fold selectivity over cathepsin B [133]. This motif was also used for affinity labeling of cathepsin L, which will be discussed in the later sections. These classes of compounds, especially N-unsubstituted aziridinyl peptides and in special cases N-acylated ones, exhibited a high selectivity and potency and provided the premise for the further development of chemical biology tools much needed for functional studies. 

### 3.6. Peptidyl Aryl Vinylsulfones

**Figure 9:**
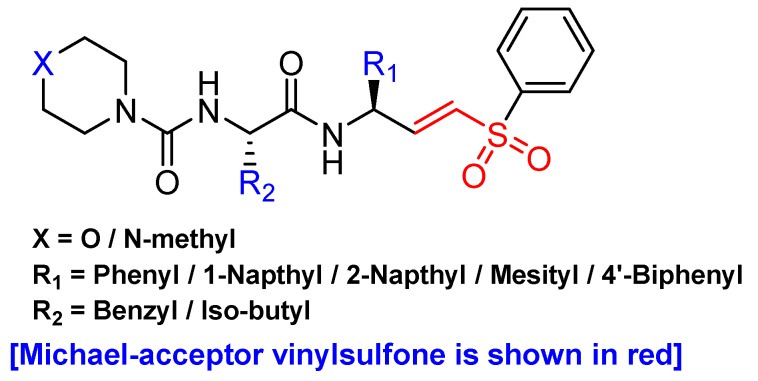
The general structure of the library of peptidyl aryl vinylsulfones where substituents (R_1_ and R_2_) have been varied to assess the favorable interactions within the cathepsin L active site.

Another promising scaffold that acts as a Michael acceptor and hijacks the catalytic residue of cysteine cathepsins is peptidyl aryl vinylsulfones. This scaffold was first introduced by Palmer et al. as an irreversible inhibitor of cysteine cathepsins [134]. Subsequently, they extended their study by performing an SAR analysis of this class of inhibitors which showed pan-cathepsin inhibition with occasional selectivity towards cathepsin S for certain scaffolds [135]. However, in a separate study by Mendieta et al., the authors developed a structurally novel library of twenty peptidyl 3-aryl vinylsulfones (Figure 9) in which they introduced extensive diversity at the R_1_ position. Subsequently, they also varied the R_2_ position while keeping either morpholine or N-methyl piperazine group intact [116]. Docking studies with most active and selective inhibitor (Entry 15, Table 2) of this class revealed that the inhibitor extends from S2 to S2′ sites of cathepsin L and the β-vinylsulfone moiety resides in a close proximity of Cys-25 residue thereby favoring the formation of Michael adduct. The authors postulated that considering the efficacy of peptidyl aryl vinyl sulfones, strong anti-cancer candidates could be harnessed by cultivating this scaffold.

### 3.7. Peptidyl Aryl Vinylsulfonate

**Figure 10:**
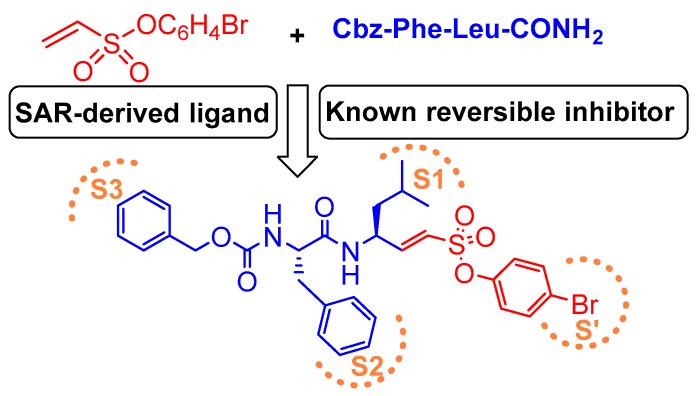
4-bromophenylvinylsulfonate moiety was tethered to a known reversible inhibitor of only modest potency to achieve a highly potent and selective irreversible peptidyl cathepsin L-selective inhibitor, KD-1; this inhibitor likely binds to both prime- and non-prime sites of the enzyme.

One other very potent class of inhibitors that also includes a Michael acceptor is peptidyl aryl vinylsulfonate esters, a superior Michael acceptor than vinyl sulfone. They served as potent inhibitors of cruzain—a parasitic cysteine protease from *T. cruzi* that is homologous to cathepsin L [136,137]. This scaffold was explored by Dana et al., who determined the superiority of aryl vinylsulfonate ester over aryl vinylsulfone and aryl vinylsulfonamide counterparts towards cathepsin L inhibition [117]. Thus, they synthesized and screened the efficacy of a library of aryl vinylsulfonate ester compounds against cathepsin L; 4-bromo phenyl vinylsulfonate was found to be the champion ligand presumably due to favorable interactions between the 4-bromo phenyl moiety with the prime site residues of cathepsin L. They further designed a hybrid inhibitor, KD-1, (Entry 16, Table 2) by strategically appending the 4-bromophenyl vinylsulfonate moiety as electrophilic warhead to a modestly potent reversible cathepsin L inhibitor [113]; this design was based on the hypothesis that the developed compound will target both the prime site and the non-prime site residues for interaction. KD-1 indeed exhibited almost a diffusion-controlled inactivation kinetics while maintaining an excellent selectivity profile toward cathepsin L (Figure 10). Furthermore, KD-1 was cell-permeable and inhibited the intracellular activity of cathepsin L in human MDA-MB-231 breast cancer cell lines. KD-1 also enhanced the integrity of cell–cell junctions by effectively attenuating the migratory potential of the cells, as demonstrated by the scratch assay. The authors anticipated that this class of inhibitors may find extensive usage in deciphering context-specific cathepsin L biology.

### 3.8. Gallinamide A Analogs

**Figure 11:**
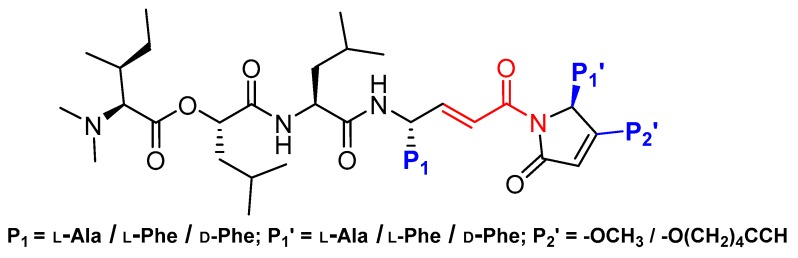
Gallinamide was reconstructed with altered amino acid sequence at P1, P1′, and P2′ positions. The acrylic group, the Michael acceptor, is shown in red.

Recently, another interesting discovery that provided a wealth of information on cathepsin L-inhibitor interactions came from marine cyanobacterial extracts. Miller et al. first reported gallinamide A as potent irreversible inhibitor of cathepsin L with an IC_50_ value of 5 nM and a 28- to 320-fold greater selectivity over cathepsin V and B, respectively [138]. They further performed molecular docking and molecular dynamics simulations and learned that the peptidyl backbone of the inhibitor spans the active site whereas the side chains engage in favorable interactions with different active site pockets, placing the Michael acceptor enamide in close proximity to the catalytic Cys residue. In a follow-up study, Boudreau et al. performed molecular docking studies to predict the potential modifications of a gallinamide A scaffold that would harness favorable enzyme–inhibitor interactions and enable the development of compounds with improved inhibitory efficacy [118]. They synthesized a panel of compounds by retaining gallinamide A and only varying the amino acids at P1, P1′, and P2′ positions. (Figure 11). This led to the discovery of the most potent analog of this series (Entry 17, Table 2) with sub-nanomolar IC_50_ value (94 pM) and fast time dependent inactivation kinetics, suggesting an improved binding and reactivity of the inhibitor with the enzyme active site. The authors found that this class of compounds effectively inactivated cruzain, a homologous cysteine protease from *T. cruzi*, using cell-based assay. Gallinamide A and its analogs thus provide a remarkable inhibitory scaffold that could potentially be harnessed to build selective enzyme inhibitors for a variety of therapeutic applications.

### 3.9. Peptidyl Aldehydes

**Figure 12:**
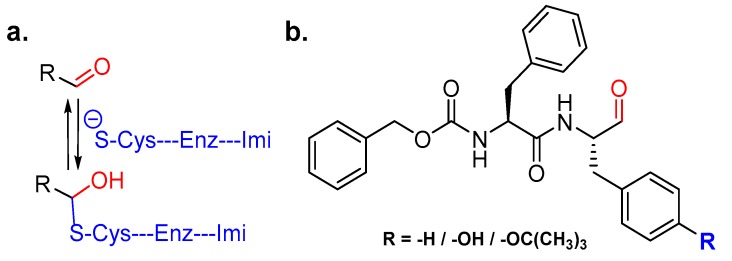
(**a**) Proposed mode of cathepsin L inactivation by aldehyde inhibitors. (**b**) Chemical structure of inhibitors, as discussed in text, are shown here.

One classical inhibitor that has been used over a long period of time to dissect cysteine proteinase activity is Leupeptin, a microbial product. Leupeptin is a peptidyl aldehyde that occupies the active site cleft of cysteine cathepsins and forms a thiohemiacetal intermediate by trapping catalytic cysteine residue; this complex hydrolyzes over time, thus showing the covalent and reversible nature of the inhibitor (Figure 12a). Leupeptin unfortunately suffers from non-specific inhibition of both serine and cysteine proteinases, thus making it unfavorable for clinical usage and chemical biology applications. This issue was, however, addressed by Woo et al. who designed and synthesized six peptidyl aldehyde analogs that were more potent than Leupeptin (IC_50_ = 70.3 nM) and exhibited improved selectivity towards cathepsin L over cathepsin B and calpain II [139]. The most potent cathepsin L inhibitor of this series was Z-Phe-Phe-H (IC_50_ = 0.74 nM) (Figure 12b) that showed more than 90-fold selectivity over cathepsin B. Interestingly; their data demonstrated the importance of aromatic amino acids, such as phenylalanine and tyrosine, at the P1 position in determining the potency and selectivity towards cathepsin L; O-alkylation of tyrosine group diminishes the inhibitory efficiency as in Z-Phe-Tyr(Bu)-H (IC_50_: 6.96 nM). In a follow-up publication, they further tested the efficacy of Z-Phe-Tyr-H (IC_50_: 0.85 nM, 100-fold selective over cathepsin B) (Figure 12b) in vitro and in vivo [140]. This compound effectively inhibited parathyroid hormone-stimulated osteoclastic bone resorption in pit formation assays, and suppressed bone weight loss of ovariectomized mouse in a dose-dependent manner when administered intraperitoneally.

**Figure 13:**
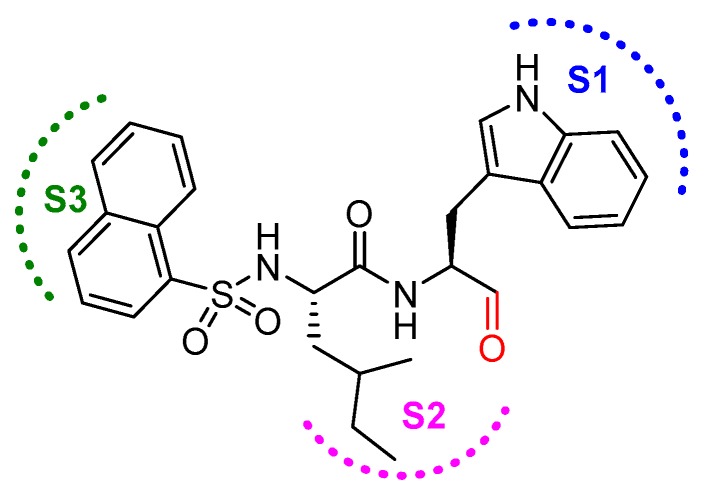
*N*-(1-naphthalenylsulfonyl-l-isoleucyl-l-tryptophanal orients itself into the active site of cathepsin L and finds favorable interactions within the S1, S2, and S3 pockets of the enzyme.

In another interesting study, Yasuma et al. developed a library of compounds by varying the amino acid substituents at P1, P2, P3 position of the inhibitor and carried out a thorough SAR study [21]. Their study revealed that the configuration of the stereogenic center (S-configuration is favored over R-configuration) at the P1 position, and not the steric factor, was key to the inhibitory efficacy. Apparently, the substituent at P1 position does not interact with S1 position residues; rather, proper stereogenicity allows the placement of the inhibitor in vicinity of the catalytic cysteine residue for interaction. S2 subsite of cathepsin L, on the other hand, preferred a hydrophobic and moderate-size group; α-branched alkyl chains but not the bulkier groups like phenylalanine was favorable. Further, the S3 subsite showed a preference for hydrophobic and bulky moieties such as 1- and 2-naphthalenylsulfonyl substituents. Among synthesized compound, *N*-(1-naphthalenylsulfonyl-l-isoleucyl-l-tryptophanal (IC_50_ = 1.9 nM, 789-fold selective over cathepsin B; Figure 13) attenuated the release of Ca^2+^ and hydroxyproline from bone in an in vitro bone culture system and further restricted bone loss in ovariectomized mice dosed orally.

A further modification of this scaffold was reported by Lynas et al. [119] Here, authors designed and developed di- and tri-peptidyl α-keto-β-aldehydes, based on substrate and inhibitor specificity profiles of cathepsin L. The compound Z-Phe-Tyr(OBut)-COCHO (Entry 18, Table 2) turned out as highly potent and selective inhibitor of cathepsin L with K_i_ value of 0.6 nM. This molecule was further adapted by Shenoy et al. to assess the structural basis for cathepsin L inhibition [96]. In their study, the authors crystallized the glyoxal inhibitor with cathepsin L; the β-aldehyde forms a tetrahedral thiohemiacetal and α-keto oxygen atom is stabilized by the oxyanion hole. The Tyr(OBut) group was found to occupy S1 site while phenyl and carboxybenzyl groups occupied S2 and S3 sites, respectively. This class of inhibitors has successfully been deployed in the functional biology of cathepsin L.

### 3.10. Azepanone-based Inhibitors

**Figure 14:**
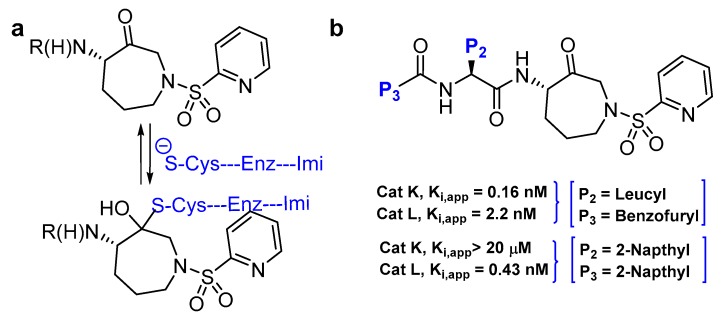
(**a**) Pictorial representation of mechanism of azepinone-mediated cathepsin L inhibition. (**b**) Generic structure of azepinone inhibitors. The inclusion of bulky aromatic groups at P2 and P3 positions leads to cathepsin L inhibitors with improved potency and selectivity.

Azepanone-based compounds were first reported as orally bioavailable and extremely potent inhibitors of cathepsin K, as shown by the pharmacokinetic studies in the rat [141]. Marquis et al. subsequently adopted the template and extended their work to acquire a selective inhibitor of cathepsin L with similar potency [120]. This class of inhibitors are armored with keto functional group that act as an electrophilic warhead and traps cysteine proteases by forming a transient covalent bond with the active-site Cys residue, rendering inactivated enzyme. The authors initiated their work by scrupulously studying the cathepsin K-inhibitor complex that revealed the influence of P2 and P3 substituents of the inhibitor in determining the efficacy and selectivity profile of the compound. Based on these observations, they designed and synthesized a series of compound and secured a highly potent cathepsin L inhibitor (K_i,app_: 0.43 nM; Entry 19, Table 2) that exerted remarkable selectivity over cathepsin K and fairly modest selectivity over both cathepsin B and S. Interestingly, SAR showed that replacement of P2 leucine and P3 benzofuran of cathepsin K inhibitor with bulkier hydrophobic aromatic groups yielded an improved potency and the selectivity towards cathepsin L (Figure 14). Molecular docking studies further supported this observation as cathepsin K was found to have a shallower S2 pocket than cathepsin L, thus incorporation of bulkier napthyl group at P2 position favored cathepsin L inhibition but not cathepsin K. On the other hand, inclusion of another napthyl group at P3 position promoted a steric clash rather than furthering the desired hydrophobic interactions within the S3 pocket of cathepsin K, thus incurring a better selectivity profile towards cathepsin L over cathepsin K. This template has proven to be an important tool to study cysteine cathepsins as it has been further extended to achieve potent cathepsin S-selective inhibitor with cellular activity [142].

### 3.11. Nitrile-Containing Inhibitors

**Figure 15:**
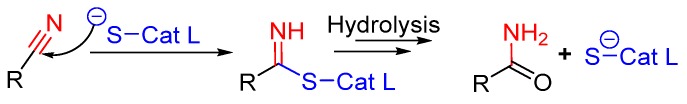
Nitrile group-containing inhibitors forms hydrolytically an unstable thioimidate bond with the catalytic cysteine residue of the enzyme.

**Figure 16:**
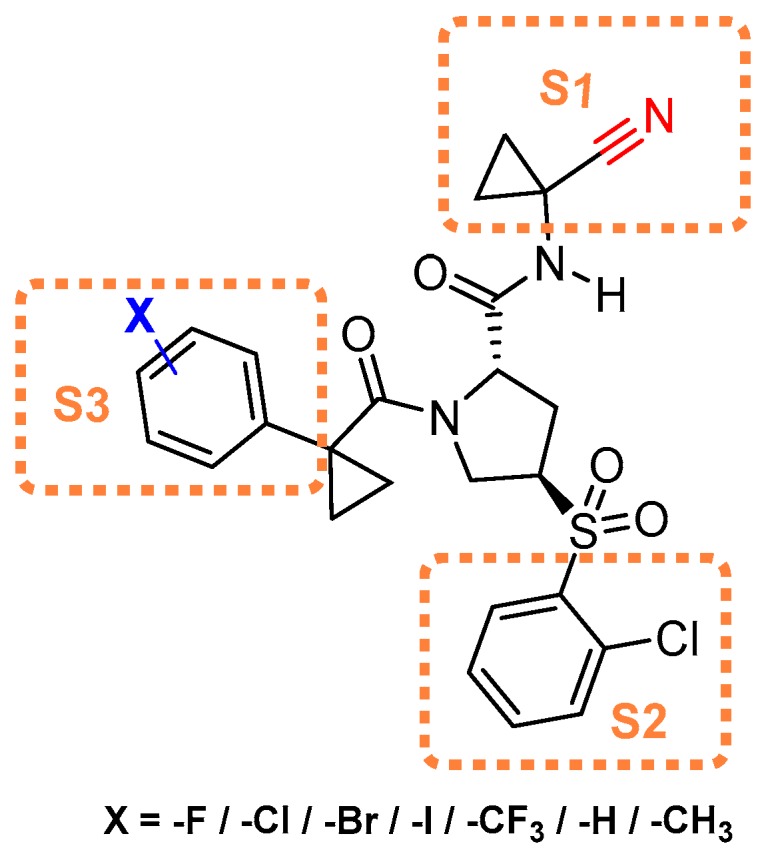
Substituted aryl groups were screened and evaluated to harness favorable halogen-protein interaction by targeting S3 pocket of cathepsin L enzyme.

Nitrile group containing inhibitors have been widely recognized as covalent and reversible inhibitors of a certain class of enzymes that depend on cysteine-mediated nucleophilic attack for catalysis; the nitrile residue traps the sulfur and forms a thioimidate bond (Figure 15) that hydrolyzes over the time rendering free enzyme. Odanacatib is one of the prime examples of this class of compounds that has been evaluated as a clinical agent, although with limited success [143,144]. Because of nitrile’s tunable target engagement nature, this scaffold has been adapted to target other relevant enzymes, including cathepsin. Hardegger et al. utilized nitrile warhead and examined the effect of halogen bonding in protein–ligand interactions [121]. They developed a series of compounds and performed a thorough SAR analysis in which the nitrile electrophile faced towards S1 site and trapped the catalytic cysteine. In the developed analogs, the substituents that occupied the S3 site were systematically varied by strategically altering the substituents at the para-position of the phenyl group (Figure 16). The authors observed an improvement in inhibition profile with the placement of halogen at the para-position of phenyl ring which followed a trend Cl < Br < I (Entry 20, Table 2), with the F substituent being an outlier. Further analysis of the enzyme-inhibitor co-crystal structures revealed that halogen at the para position of the phenyl ring suitably interacted with Gly61 at the S3 site; fluorine analog pointed away to avoid the electronic repulsion from the oxygen lone pairs of Gly61. The authors have also performed computational analysis which taken together with the crystal data suggests O· X-C angle and the distance between the interacting atoms primarily influenced the protein-ligand interaction. This work provides an important roadmap for developing improved chemical biology tools where a halogen-protein interaction has successfully been utilized [97]. 

**Figure 17:**
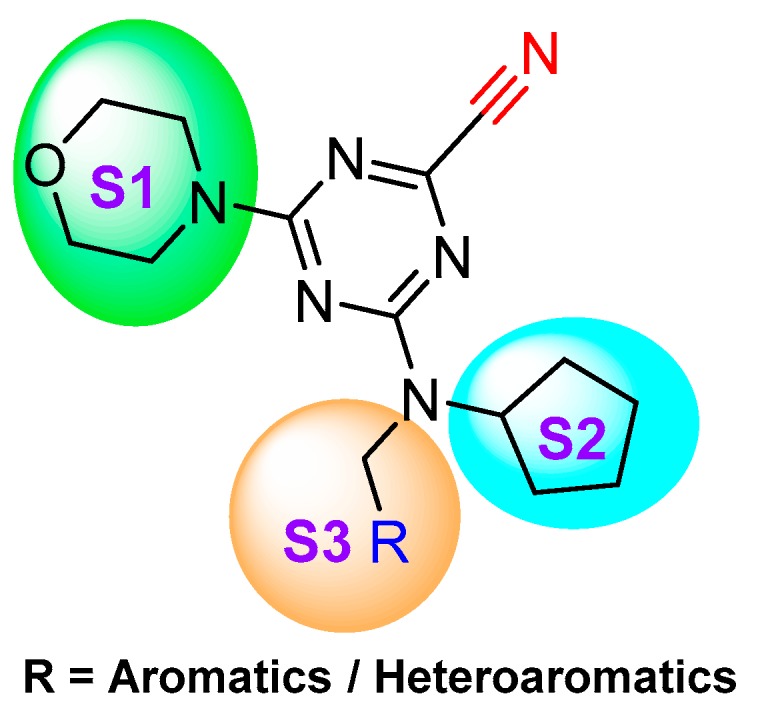
Triazine nitrile scaffold was utilized with varying substituents that explored the S3 pocket on the enzyme for cathepsin L inhibition.

To examine what effect amide···heteroarene π-stacking interactions may have on chalcogen bonding in the S3 pocket of cathepsin L, Giroud et al. utilized triazine-nitrile scaffold [145]. The authors synthesized a diverse set of triazine-nitrile compounds with a diversified heteroarenes targeting S3 pocket; the S1 and S2 substituents were kept constant. Among the developed compound library, 2-benzothienyl analog (Entry 21, Table 2) exhibited maximum inhibitory potential; 2-benzofuranyl, 2-benzothiazolyl, and 2-imidazopyridinyl, which are of similar geometry, also followed a similar inhibitory pattern (Figure 17). Molecular modelling based on co-crystal structures showed favorable chalcogen interaction to the backbone carbonyl of Asn66 (d(S···O = CAsn66) = 3.5 Å and the angle α(OAsn66···S-C) = 158°) at the S3 pocket; this was further supported by a conformational strain analysis, as chalcogen–enzyme interactions compensated for higher torsional strain in the S-containing ligands when compared to the benzofuranyl and imidazopyridinyl ligands. Their study demonstrated the importance of both intermolecular interactions and conformational strain in assessing the effect of heterobicyclic ligands at the S3 pocket that could be potentially be utilized to develop cathepsin L selective inhibitors.

**Figure 18:**
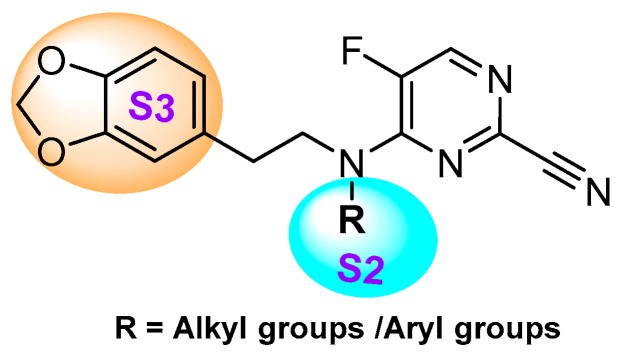
Diverse set of pyrimidine-based nitrile Inhibitors, with ligands targeting the S2 pocket of cathepsin L for inhibition.

In a subsequent study, Kuhn et al. systematically compared the effectiveness of four different approaches: (a) selection by a medicinal chemist (b) manual modeling (c) docking followed by manual filtering, and (d) free energy calculations (FEP). This systematic protocol enabled them to prioritize building blocks for effective targeting of cathepsin L enzyme [123]. The authors developed a series of 36 analogs by varying only S2 substituents and keeping S1 and S3 fixed (Figure 18). After analyzing the affinity by enzyme kinetics, they found that the FEP method was superior over other well-established methodologies; this method not only predicted the most relevant ligands but also identified the topological requirements of the substituents for a more effective engagement in the S2 pocket. Among the developed compounds, cyclopentylmethyl substituent in the S2 pocket (Entry 22, Table 2) incurred the most favorable interaction as it optimally filled the front part of the pocket. This strategy certainly provided an edge over other conventional methodologies in predicting the optimal ligands for the S2 pocket targeting. These findings could benefit the ongoing effort of achieving a suitable therapeutic candidate for cathepsin L enzyme.

The readers are encouraged to refer to studies by Falgueyret et al. and Asaad et al. where, although not selective, nitrile-containing inhibitors targeting cathepsin L have also successfully been reported [146,147].

### 3.12. Thiosemicarbazone

**Figure 19:**
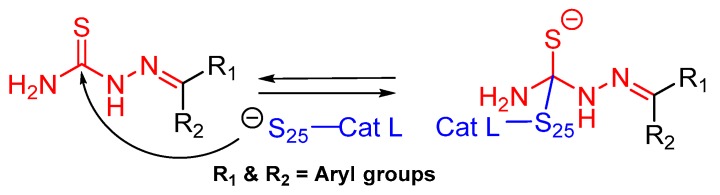
Catalytic cysteine residue of cathepsin L forms a covalent and reversible bond with the thiosemicarbazone group rendering the inhibited enzyme.

The thiosemicarbazone moiety was first recognized as a relevant covalent and reversible warhead of cathepsin L homologous enzyme cruzain, a protease from *T. Cruzi*. The mechanism of inactivaction involves the formation of a transient covalent bond with the catalytic Cys residue (Figure 19) [148]. In an interesting study, Kishore Kumar et al. first utilized this idea and synthesized a small library of compounds in which the most active class of inhibitors were comprised of one meta-bromo substituted aryl ring along with another one with optimally substituted functionalities [127]. The inhibitor places itself in the active site cleft of cathepsin L where meta-bromo substituted aryl ring occupies the S2 site and thiosemicarbazone motif lies near the active site cysteine.

However, when the motif was extended to capture S1′ site interaction by placing the aryl/alkyl group at the terminal nitrogen of thiosemicarbazone, the inhibitory potency was completely diminished. Overall, this class of inhibitors showed a good selectivity over cathepsin B and exhibited low cytotoxicity when tested on human cancer cell lines. In follow up studies, Kishore et al. and Parker et al. further expanded the scope of thiosemicarbazone scaffold and developed diversely functionalized analogs that exhibited an enhanced inhibitory potency and promising cellular activities while still retaining the selectivity over cathepsin B [125,126]. In their latest study, Parker et al. strategically transformed an active inhibitor with limited aqueous solubility into a water-soluble prodrug (Entry 23, Table 2), by phosphorylation of phenolic hydroxy group; this group was readily hydrolyzable by alkaline phosphatases, rendering the active pharmacophore [124]. The phosphate prodrug exhibited a remarkable 600-fold increase in solubility over the parent drug and did not disintegrate in aqueous solution, even after prolonged exposure at the physiological temperature. Furthermore, this compound did not show any significant cytotoxicity on normal primary HUVECs cells in comparison to other FDA-approved cytotoxic drugs, Doxorubicin and Paclitaxel. This prodrug thus far has shown promise to be a desirable clinical candidate and the authors have proposed to evaluate its in vivo efficacy in a preclinical setup.

### 3.13. Propeptide Mimics 

As noted earlier, cathepsin L, like other cysteine cathepsins, contains an inhibitory propeptide domain that spans the active site of the enzyme in the inverse direction to the regular substrate binding mode. Chowdhury et al., in their seminal study, exploited this concept by examining the effect of a series of synthesized tripeptidyl compounds that mimicked cathepsin L inhibitory propeptide [95]. Importantly, the developed tripeptidyl motifs also exhibited nanomolar potency; however, a moderate truncation of the full-length propeptide drastically lost all activities [47]. Notably, while the full-length propeptide showed only 2-fold selectivity over cathepsin K, the most potent analog of this series (Entry 24, Table 2) demonstrated a far-improved selectivity (310-fold). The authors further investigated the binding mode of this class of inhibitors by means of co-crystal structure and molecular modeling. This revealed that (a) arginine residue of the inhibitor occupied the S1 pocket, (b) phenyl alanine residue found favorable hydrophobic interactions within the S2 pocket, (c) 2-phenylethyl group pointed toward S3 pocket, (d) the methionine residue showed optimal interaction within S1′ pocket, and (e) the biphenyl acetyl group extended to the S3′ pocket for favorable interactions [95,128]. This class of inhibitors has shown resistance to enzyme-dependent hydrolysis and demonstrates the reversible mode of enzyme inactivation. Overall, this inhibitor class provides a wealth of information on inhibitor binding to cathepsin L and provides a general template for the development of therapeutic candidates for other relevant enzymes as well.

### 3.14. Thiocarbazate, Oxocarbazate and Azapeptides

**Figure 20:**
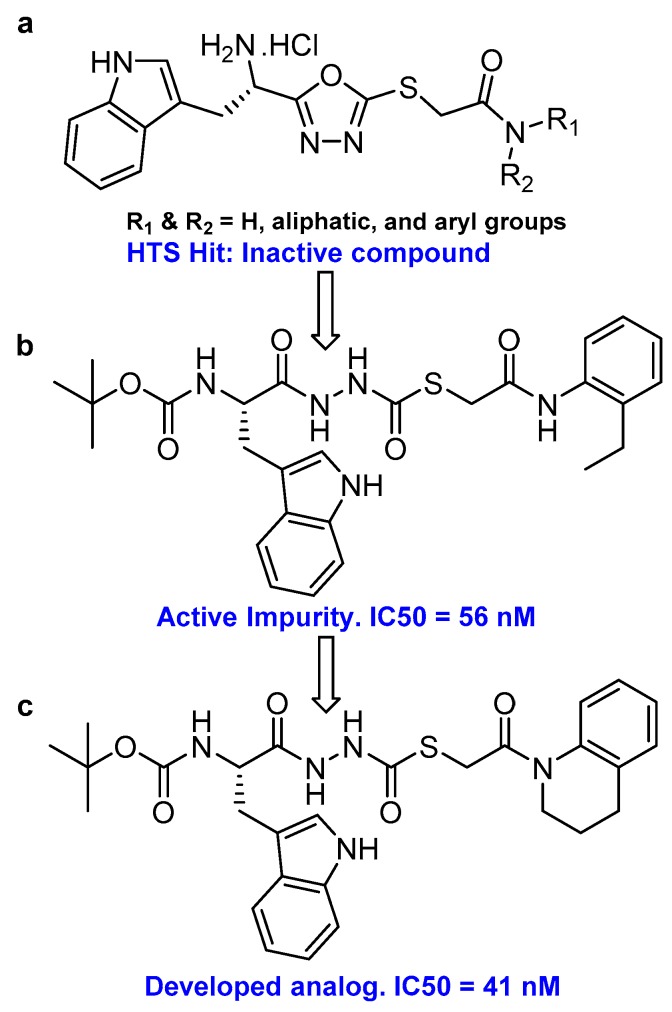
(**a**) Structure of HTS hit that was a false positive. (**b**) Structure of the impurity that turned out to be an active pharmacophore. (**c**) The most potent analog of this series, achieved by SAR analysis.

In an effort to discover small molecule inhibitors of cathepsin L, Myers et al. performed high throughput screening (HTS) of the NIH Molecular Libraries Small Molecule Repository (MLSMR); they identified 2,5-disubstituted oxadiazoles (Figure 20a) as potent hit compounds [129]. Surprisingly, upon synthesis and purification of the putative inhibitory lead compounds, a complete loss of activity was observed. The authors then investigated the compounds’ integrity from NIH MLSMR library by LC-MS; this showed the presence of additional impurities. To trace back the active impurity, the authors hypothesized the presence of impurities resulting from an acid-catalyzed ring-opening reaction of thiocarbazate. The resulting azapeptides was likely the active pharmacophore that inhibited enzyme via acylation of active site Cys; this was validated by synthesis of azapeptides and performing the enzyme assay [149]. The (*S*)-stereoisomer of newly synthesized compound (Entry 25, Table 2; Figure 20b) indeed attenuated the activity of cathepsin L with an IC_50_ value of 56 nM. In follow up studies, the authors further developed a series of compounds with structural diversity and performed a computational analysis to recognize the basis of potent enzyme inhibition [130,150] One of the thiocarbazate analogs developed this way (Figure 20c) showed improved potency over the parent compound. Molecular modeling studies performed with parent compound (Entry 25, Table 2) in complex with papain indicated that indole motif preferably bound to S2 subsite, -NHBoc group engaged in favorable hydrophobic interactions within the S3 subsite and 2-ethylphenyl anilide extended to S1′ pocket. To further probe the importance of thiocarbazate moiety, the authors synthesized compound containing oxocarbazate (Entry 26, Table 2) and azapeptide (Entry 27, Table 2) motifs. The oxocarbazate showed a fairly improved IC_50_ value (7 nM) towards cathepsin L, whereas the azapeptide was at best only a modest inhibitor (IC_50_ = 3 µM). Consistently, the binding mode of oxocarbazate exerted similarity to that of thiocarbazate when investigated by molecular modeling studies [150].

In a separate study, Shah et al. carried out a thorough enzymatic analysis of the champion thiocarbazate compound (Entry 25, Table 2) that showed a time-dependent improvement in the inhibition profile; the IC_50_ value went down to 1 nM when preincubated with cathepsin L for 4 h [131]. LC-MS and kinetic analysis of enzyme-inhibitor complex (inhibition rate constants: k_on_ = 24,000 M^−1^s^−1^ and k_off_ = 2.2 × 10^−5^ s^−1^, and binding constant: K_i_ = 0.89 nM) demonstrated a slow-binding kinetics and reversibility of inhibition. The selectivity over other members of the enzyme family was modest. Interestingly, the compound inhibited propagation of malaria parasite *Plasmodium falciparum* [IC_50_ = 15.4 µM], and *Leishmania major* [IC_50_ = 12.5 µM], and did not exhibit any significant toxicity against human aortic endothelial cells and zebrafish. Although thiocarbazate motif showed promise as an inhibitory scaffold, the lack of reasonable stability (it decomposes even in DMSO) and only modest inhibitory activity in cell-based assays probably ceased any further development of the scaffold [129]. The authors also extended their studies to evaluate the potential of oxocarbazate inhibitor that showed an improved IC_50_ value of 0.4 nM upon 4 h preincubation with the enzyme. Like as in the case of thiocarbazate, they performed an enzyme kinetic analysis of the enzyme-inhibitor complex and obtained the following parameters: inhibition rate constants: k_on_ = 153,000 M^−1^s^−1^ and k_off_ = 4.4 × 10^−5^ s^−1^, and binding constant: K_i_ = 0.29 nM [132]. The inhibitor blocked SARS-CoV (IC_50_ = 273 ± 49 nM) and Ebola virus (IC50 = 193 ± 39 nM) entry into the human embryonic kidney (HEK) 293T cells, a process that utilizes cathepsin L-mediated proteolysis for host cell infection. The oxocarbazate, when treated with HEK 293T lysate in the presence of DCG-04, an activity-based cysteine cathepsin probe, showed reduced cathepsin L labeling when assessed by a Western-blot analysis; this further corroborated the results obtained from the virus pseudotype infection assay. Overall, oxocarbazate inhibitor not only provided a promising template for further exploitation but also rendered a new direction for intervening SARS and Ebola virus infections.

## 4. Molecular Probes

Ubiquitous expression of human cathepsin L in most human tissues possesses a significant challenge in targeting this enzyme for therapeutic development. This problem is further exacerbated with recent findings that alternative spliced isoforms could exist at distinct cellular locations (e.g., nucleus, cytosol, and ECM space) [37,38,151]. While several unique functional roles of cathepsin L are known, it has also been reported that some of its function can also be accomplished by other members of the cathepsin family (i.e., functional redundancy); for example, both cathepsin L and B can mediate a mutually compensatory role in the inflammatory response signaling pathways [152]. In this regard, accurate function of cathepsin L must be first determined in different cell types individually before considering significant investment in drug development. Since the function of an enzyme, such as cathepsin L, depends primarily on its activity profile and given that the activity profiles of differentially processed cathepsin L isoforms may be very different, probes capable of reporting accurate activity status in different cell types (and cellular location) are anticipated to advance our understanding if cathepsin L biology. Over the years, the concept of Activity-Based Probes (ABPs) has emerged as a valuable chemical biology tool for monitoring the enzyme activity (not just the expression profile alone) in cells at the proteome levels [153,154,155,156].

**Figure 21:**
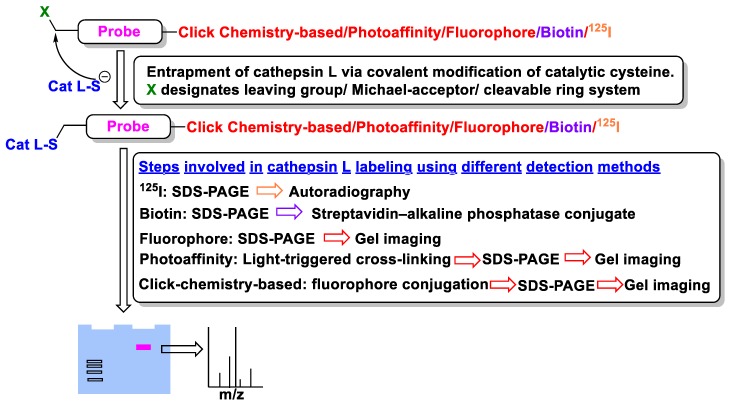
Schematic representation of enzyme-labeling and proteome-wide activity profiling studies using probes for selective detection of active and functional cathepsin L enzyme.

In most cases, existing covalent inhibitors containing a recognition motif are adopted and transformed to ABPs by conjugating optimal detection modalities; these include fluorescent probes, affinity labels, radiotracer, and many others (Figure 21). Indeed, the use of ABPs has rather established cathepsins as key diagnostic marker for various disease conditions, and have even enabled optical surgical navigation, leading to an improved surgical precision [157,158]. In the following sections, we discussed cathepsin L-selective molecular probes that have been developed and utilized for monitoring its activity. A thorough overview of cathepsin probe development for imaging purpose could be found elsewhere [10,159,160,161].

### 4.1. Radio-Labelled

Radio-labeled inhibitors have long been used as a primary mode for detecting active cysteine proteases both in vitro and in vivo. Docherty et al. first used a chloromethyl inhibitor containing radioactive iodine, ^125^I-Tyr-Ala-Lys-ArgCH_2_Cl, and detected cathepsin B in crude granule fraction of islet cells [162]. In their follow up study, they also presumably identified cathepsin L in insulin secretory granule using the same radio-isotopically labeled inhibitor [163]. The mechanism of detection involves covalent modification of the target protein that shows up as a distinct band upon performing autoradiography. This chloromethyl containing inhibitor turned out to be non-selective due to its reactivity towards trypsin, a serine protease. This was followed by the discovery of a radioactive-peptidyldiazomethane compound (Entry P1, Table 3), a selective cysteine proteinase inhibitor [164]. Mason et al. adopted the peptidyldiazomethane scaffold, which potently inhibited both cathepsin L and B. This scaffold showed improved inhibition profile upon iodination, as demonstrated by Crawford et al. [103] This inhibitory agent was then transformed to a radio-labeled probe via incorporation of ^125^I [164]. The developed probe was utilized to detect both cathepsin L and B in Kirsten-virus-transformed KNIH 3T3 cells. The incubation of cellular extracts with P1 followed by gel electrophoresis showed the presence of two protein bands at 30 and 23 kDa, showing two active forms of cathepsin L. Active cathepsin B was also detected at around 33–35 kDa. Interestingly, pulse-chase experiments with [^35^S]methionine-labeled proteins only detected two separate bands at 36 kDa and 39 kDa, which correspond to the intracellular inactive precursors of cathepsin L and B respectively. This indicated that the inactive precursor proteins did not react with P1, demonstrating its unique ability to quantify only active protein. Further, P1 was utilized to probe active cathepsin L and B in different human tissues as well as in lysosomes and whole cells [165,166]. In a follow-up study, Xing et al. developed Fmoc-[I_2_]Tyr-Ala-CHN_2_ (Entry P2, Table 3) that selectively detected cathepsin L and B over cathepsin S, exhibiting a faster rate of inactivation towards cathepsin L [167]. The developed compound successfully probed the amount of active cathepsin L and B in different cell-lines; two unknown proteins also got labeled in certain cases. Overall, these probes enabled the detection of active cathepsin enzymes with their cellular location, thereby advancing the knowledge of cathepsin L biology.

### 4.2. Affinity-Based

Gelhaus et al. first developed biotinylated aziridine-2,3-dicarboxylate and demonstrated its anti-plasmodial activity using cell-based studies [168]. Since, the biotinylated compound inhibited plasmodial protease falcipain and cathepsin L, authors suggested that this scaffold could be utilized for the development of cell-permeable, non-radioactive reagents that selectively labels enzymes involved in parasite pathogenicity. Later on, Vicik et al. adopted this motif (Entry P3, Table 3) and developed an affinity label to probe cathepsin L activity [133]. The aziridine analog irreversibly inactivates the enzyme via covalent modification, as discussed previously. The conjugated biotin moiety is utilized for affinity pull down and target identification. When (*S*,*S*) isomer of the biotinylated probe was incubated with cathepsin L and subjected to gel electrophoresis, electro-transferred to a membrane, and exposed to streptavidin-alkaline phosphatase conjugate, a strong labeling of the enzyme-inhibitor complex was observed. However, when the enzyme was treated with E-64, an active site-directed competitive and irreversible cathepsin inhibitor, prior to incubation with P3, the labeling was diminished, clearly demonstrating that P3 competes for the active site of cathepsin L. In line with this observation, a desthiobiotinylated analog also exhibited the same trend but with reduced labeling due to its weaker binding affinity to streptavidin. Although P3 has a modest binding affinity (K_i_ = 1.4 µM) to cathepsin L, it exerted a 36-fold selectivity over cathepsin B. Certainly, the affinity labeling technique not only served as an ABP for cathepsin L but also provides a premise for developing aziridine-based chemical tools for functional proteomics.

### 4.3. Photoaffinity-Based

Although covalent modifiers of proteins have been vastly exploited as chemical tools for target identification and functional proteomics, photoaffinity probes offer unique mode of action. They bind proteins non-covalently (affinity based solely on non-covalent interactions) first and form a non-selective covalent bond with the closest amino acid residue only upon irradiation. Torkar et al. took advantage of this technique and developed the first photoaffinity-based probe (Entry P3, Table 3) to detect active cathepsin L selectively over other members of the family [170]. The photoaffinity-based probe was designed based on existing peptidyl acetyloxymethyl ketone (AOMK), a known covalent modifier of cysteine proteases. During the design, the AOMK group at the C-terminus was replaced by a short di(ethylene glycol) moiety that increased the aqueous solubility and altered the character of the inhibitor from irreversible to a reversible one. The probe was comprised of a lysine residue that was strategically placed to append fluorescent cyanine-3 (Cy3) group for detection. A photoactivatable benzoylphenylalanine amino acid was placed to accommodate the S2 pocket of cathepsin L. The developed probe detected recombinant cathepsin L upon incubation and subsequent irradiation for 40 min at 365 nm as demonstrated by SDS-PAGE. The protein band became invisible when enzyme was incubated with known cathepsin L inhibitors, E-64 and GB111-NH_2_, respectively, prior to the probe treatment [175,176]. The probe also showed preferential selectivity towards cathepsin L when compared to other two fluorescent-based probes. The probe P3 exhibited a remarkable selectivity for cathepsin L over all other cathepsins in light-mediated labeling experiments with recombinant proteins. The relative labeling percentages (cathepsin B and K: 4%; cathepsin S: 1%) were insignificant, except for cathepsin V (27%), the closest homolog of cathepsin L, relative to cathepsin L. Interestingly, U87-MG glioma cell extracts did not present any cathepsin L for detection with the P3 probe. However, when the same cell extracts were treated with recombinant cathepsin L added externally and subjected to a labeling experiment, the probe selectively detected the desired protein in complex proteome. The mechanism of protein labeling by P3 was attributed to putative bond formation between benzophenone and non-conserved Met161 at the S2 site of cathepsin L. Although the developed probe lacks cell-penetrability, the authors envisioned that the technique might have diagnostic and prognostic value where cathepsin L overexpression is high, such as in malignant tissues. 

### 4.4. Fluorescence-Based

#### 4.4.1. Two-Photon Based

Although several fluorescent-based molecular probes have been reported for cysteine cathepsins, the superiority of two-photon-based over one-photon based imaging technique inspired Na et al. to develop probes with a better cellular imaging profile [171]. Notably, the two-photon fluorescence imaging technique provides increased tissue penetration depth with reduced photobleaching, and a lower tissue autofluorescence. The authors first fabricated a microarray with 105 different peptidyl aldehydes and screened against GFP-labeled cathepsin L enzyme; this led to the identification of two inhibitory hits that potently inactivated enzyme with IC_50_ values of 14.5 and 20.8 nM. The lead inhibitors were further structurally modified to include (a) a two-photon dye, DL-1 (a 4,6-bis(4-hydroxystyryl)pyrimidine derivative), at P1 position, and (b) Disperse Red 1 dye, a fluorescence quencher, in the place of aldehyde moiety. The resulting imaging probes (Entry P5 in Table 3 is one of such examples) showed a time-dependent increase in fluorescence signals when treated with HepG2 cell lysates, a mammalian liver cancer cell-line known to overexpresses cysteine cathepsins. Enhanced fluorescence signal is due to the release of the quencher upon successful proteolytic cleavage. Furthermore, to assess the suitability of P5 as imaging agents for live cells, HepG2 cells were incubated with the probe and subjected to a live-cell imaging analysis. There were strong fluorescence signals from endolysomal compartments that disappeared completely when cells were pretreated with E-64, validating the target specificity of the probe. As the probe was developed using cathepsin L as a model enzyme, it likely will lack specificity and perhaps interact with other members of the family. Still, however, this motif could potentially be utilized to develop selective ABPs targeting respective cathepsins and assess their activities in a tissue environment, as proposed by the authors.

#### 4.4.2. One-Photon Based

Activity-based probes with a single photon fluorescent tag have been successfully utilized for the functional analysis of target proteins both in vitro and in vivo. Poreba et al., in their pursuit of developing cathepsin L selective imaging agent, took advantage of this technology [172]. They realized the importance of developing selective cathepsin L substrate that will bind to the active site of the enzyme over other homologous proteins. To do so, they employed Hybrid Combinatorial Substrate Library (HyCoSuL) technology that provides information on optimal chemical space inside the enzyme active sites by strategically scanning diverse peptide library containing both natural and unnatural amino acids. This led to the acquisition of a panel of compounds with desired properties. The potent hits discovered by HyCoSuL technology were further optimized to gain selectivity over other cathepsins while identifying the most efficient substrate based on Michaelis Menten parameters. Unfortunately, when the chosen peptide substrate transformed to an activity-based probe by appending an acyloxymethylketone warhead and a biotin tag, it showed cross-reactivity with cathepsin B. Despite biotinylated probe’s somewhat compromised selectivity, the authors still assessed its activity-based labeling profile in HEK293T cells; as expected, cathepsin L labeling was primarily observed with minor amounts of cathepsin B. Further optimization by swapping the biotin and Arg with cyanine-5 and Cys(Bzl) (Entry P6, Table 3) groups was carried out next. The newly developed fluorescent ABP, P6, showed an enhanced selectivity (in comparison to pan-cathepsin probes) against a panel of other recombinant cathepsins (cathepsin V, B, S, and K), as well as cellular extracts derived from HEK293T and MDA-MB-231 cells. The developed probe served as an effective imaging agent for cellular cathepsin L activity in human MDA-MB-231 breast cancer cells when incubated for 8 h. The selectivity of the probe started to recede with longer incubation time. The importance of optimizing the probe concentration and time-course of the reaction was evident from these experiments. Interestingly, P6 only detected active cathepsin L and not procathepsin L; this certainly signifies the effectiveness of the developed probes as an activity-based probe. The authors further examined the colocalization of the probe with both cathepsin L and B in MDA-MB-231 cells by treating the cells with respective cathepsin antibodies and performing a quantitative pixel analysis from a set of fluorescence microscopy images. This supported the previous observation as the highest weighted colocalization coefficient was obtained for cathepsin L and not for cathepsin B. The developed compound certainly harbors the key traits of an effective activity-based probe, as the authors duly envisioned its significance in deciphering cathepsin L biology in the coming years.

### 4.5. Clickable and Tagless 

As noted above, fluorescence-based imaging probes have been successfully developed to gain access to unknown functionalities of cathepsin enzymes. However, the bulkiness and often multiple charges associated with the fluorophore and/or quencher structures on these probes likely render them poorly cell-permeable and reduce their target affinity. To address this issue, Dana et al. adopted a previously reported peptidyl vinylsulfonate inhibitor KD-1—a highly potent, selective, covalent and irreversible inhibitor of cathepsin L discussed in Section 3.7—and tactically appended a small alkynyl group at the *para*-position of the Cbz group [117]. This led to the development of a clickable and tagless activity-based probe (catABP) of cathepsin L that retained the key desirable traits of KD-1; i.e., cell permeability, charge profile, molecular weight, potency and selectivity profile [170]. This strategy eliminated the requirements of including bulky and charged fluorophore/quencher moiety to the probe. One of the inherent advantages of this approach is that the labeling can be performed in live cellular environment with high efficiency. After cell lysis, the labeled cathepsin L can be quantified by performing click chemistry protocol with a fluorescent azide containing dye, resolving the protein using gel-electrophoresis, and directly scanning the gel for fluorescence signal. As anticipated, the developed KDP-1 probe (Entry P7, Table 3), exhibited rapid inactivation kinetics, retained selectivity for cathepsin L, and labeled recombinant active cathepsin L in an activity-dependent manner; the heat-denatured and E-64 treated cathepsin L showed no labeling when subjected to the identical labeling protocol. A mass-spectrometric analysis of the enzyme-probe complex concluded that the probe was active-site directed, and covalently modified the catalytic Cys residue for inactivation. Since the KDP-1 probe was developed with the intention of capturing active cathepsin L in vivo and in human live cell culture, the probe was tested for its cytotoxicity in MDA-MB-231 cells; no cytotoxicity was observed in these cells, even at a concentration as high as 10 µM. Incubation of MDA-MB-231 cells overexpressing cathepsin L with KDP-1 attenuated the intracellular cathepsin L activity in a dose-dependent manner; this was demonstrated by live-cell imaging and wound healing assay. Finally, KDP-1 also interfered with the hatching process of post-fertilized zebrafish embryos, further validating probe’s in vivo activity; notably, cathepsin L serves as the key regulator of the hatching process [177,178,179]. In conclusion, KDP-1 demonstrated many desirable attributes of a good probe and is anticipated to find extensive applications in probing cathepsin L function in cells from diverse origins.

### 4.6. Mass Cytometry-Compatible Activity-Based Probes

Although fluorophores-containing molecules have been appreciated as useful imaging probes, commonly used fluorophores often suffer from spectral overlapping that limits the number of targets that can be analyzed concomitantly [180]. To address this issue, Poreba et al. recently developed protease-selective lanthanide-labeled probes compatible with mass cytometry which allows subsequent analysis by both mass and imaging mass cytometry (IMC) [171]. These metal-tagged, time of flight activity-based probes (TOF) allowed them to determine cellular activities and location of three lysosomal proteases. Thus, using cathepsin L, B, and legumain as the model systems, they elegantly crafted an activity-based probe by incorporating (a) a protease-selective peptide sequence for specific enzyme recognition, (b) the acyloxymethylketone as electrophilic warhead to trap the target enzyme, and (c) the dodecanetetraacetic acid (DOTA) for metal chelation that is tethered to the peptide sequence via a linker (Entry P8, Table 3). They further incorporated three different lanthanides, ^159^Tb, ^175^Lu, and Gd (a mixture of naturally occurring six isotopes), to validate their approach and to further evaluate the influence of isotopes on enzyme binding specificity. The newly developed probes exerted promising selectivity toward both recombinant proteases and proteases from cancer cell lines, HCT-116 and MDA-MB-231.The authors were able to simultaneous detect the activities of proteases in HCT-116 cells. Moreover, each of the protease-specific probes exerted a similar labelling efficiency in HCT-116 cells, regardless of their metal counterparts, which further reinforces the compatibility of this class of probes as cytometry-labelling agents. They extended their investigation to THP-1 cells, a non-adherent monocyte-like cell line, that expresses both cathepsin B and L but contains very low levels of legumain enzyme. Probe treated THP-1 showed a clear labeling of both cathepsin B and L with no detectable activity of legumain. This finding was consistent with their transcriptional data. Finally, the developed probes not only allowed to detect the activome of cathepsin L, B, and legumain in peripheral blood mononuclear cells (PBMC) but also enabled to categorize NK cells in two distinct populations based on protease activome levels. This strategy, unlike many existing technologies, thus allowed the simultaneous detection of target proteases, thereby providing a more holistic understanding of the activome. This certainly merits the future development of TOF-based probes for multiplexed enzyme activity detection.

## 5. Final Perspectives

The inhibition of cathepsin L has continue to emerge at the forefront of drug development for several human diseases. Yet, no inhibitory agents targeting cathepsin L have advanced to clinical trials. While inhibitors of cathepsin B and S are currently being evaluated in clinical trials, recent failure of Odanacatib, a cathepsin K inhibitor for osteoporosis, in late stage clinical trial has made pharmaceutical industries wary of targeting cathepsins. The key challenge remains gaining inhibitor selectivity with respect to the other members of cathepsins and directing them to the targeted cell types for selective functional perturbation. With its ubiquitous expression profile, the function of cathepsin L in individual cell types must be precisely defined first; this is especially important since several isoforms of cathepsin L have been reported in the distinct cellular locations, and their activity and functional profile in individual cell types still remain poorly documented. While recently developed cathepsin inhibitors and probes have significantly advanced our understanding of cathepsin L function in both normal and disease cells, more efforts are needed for the development of isoform-selective reagents for further advancement; perhaps new allosteric modules on cathepsin L enzyme can be explored and exploited for precision targeting. Fortunately, several structural coordinates for cathepsin L enzyme forms, some with a diverse set of complexing ligands and some without, are now available (Table 4). These could aid in the development of isoform-selective inhibitory probes that will enable researchers to assess the context-specific needs of targeting cathepsin L in different cellular states. 

In recent years, disease-associated protease activatable prodrugs have gained much recognition and garnered breakthrough therapies in the area of antibody-drug conjugation (ADC) [181]. The majority of the ADCs are constructed by tethering a cytotoxic drug with an antibody via a protease-sensitive module for targeted delivery. Cathepsin B-specific module, for example, has been successfully implemented for this role, which led to the development of FDA approved therapies; for example, Brentuximab vedotin (Adcetris^®^) for CD30-positive relapsed or refractory Hodgkin′s lymphoma. In addition, protease cleavable prodrug strategy has also inspired the development of cathepsin B selective probe and even enabled real-time monitoring of drug release [182,183]. Interestingly, Ueki et al. slightly maneuvered this approach to acquire a prodrug which gets serially activated by histone deacetylase (HDAC) and cathepsin L, and subsequently delivers the cytotoxic payload, puromycin, to cancer cells [184]. This strategy has thus enabled selective targeting of cancer cells, specifically with high HDAC and cathepsin L activities. Taken together, these developments surely lay the foundation for the development of future cathepsin L-based therapies. Much excitement remains as new cathepsin L functions continue to emerge from specific cell types in the coming years.

## Figures and Tables

**Table 1:** Reported physiological inhibitory ligands of human cathepsin L with their inhibition constants.

Inhibitory Ligand	Inhibition Constant (K_i_)
Recombinant Cathepsin L Propeptide	0.088 nM [47] (pH = 5.5)
Cystatin A	1.3 nM [48,49]
Cystatin B	0.23 nM [48,49]
Cystatin C	<0.005 nM [50]
Cystatin D	18 nM [51]
P41 of MHC Class II Molecule	2 pM [52]
L-Kinenogen	1.7 pM [53,54]
Cystatin F	0.31 nM [55]
Sialostatin L	95 pM [45]
Antimicrobial Peptide LL-37	150 nM [56]

**Table 2:** Reported small molecule inhibitory agents of human cathepsin L, their mechanism of inhibition, inhibitory parameters and selectivity profile, and their demonstrated utilities.

#	Chemical Structure	Inhibitor Class	Inhibitor Efficacy (Cathepsin L)	Selectivity Factor ^#^	Mechanism of Inhibition (Demonstrated Utilities)	References
1	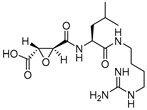	Epoxy Succinyl	2nd Order (M^−1^ s^−1^) 96250	Cat B: 1.1 Cat H: 24	Irreversible (In vitro)	Barrett et al., 1982 [99]
2	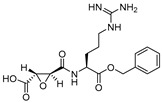	Epoxy Succinyl	2nd Order (10^3^ M^−1^ s^−1^) 73	Cat S: 89	Irreversible (In vitro)	Gour-Salin et al., 1994 [111]
3	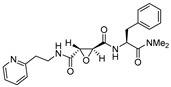	Epoxy Succinyl	10^−6^ M (100% inhibition)	Cat B: NI Cat S: 3 Cat K: NI	Irreversible (In vitro cellular)	Katunuma et al.,1999 [101]
4	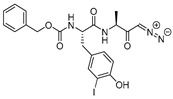	Peptidyldiazomethane	2nd Order (M^−1^ s^−1^) 1128000	Cat B: 41 Calpain: NI	Irreversible (In vitro)	Crawford et al., 1988 [103]
5	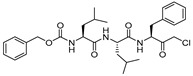	Peptidylchloromethane (chloromethylketone)	2nd Order (M^−1^ s^−1^) 21500000	Cat B: 113 Calpain: <200	Irreversible (In vitro)	Crawford et al., 1988 [103]
6	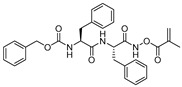	N-peptidyl-O-acyl hydroxylamines	2nd Order (M^−1^ s^−1^) 1222000	Cat B: 436 Cat S: 58 Cat H: ND	Irreversible (In vitro)	Bromme et al., 1989 [108]
7	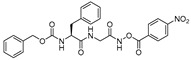	N-peptidyl-O-carbamoyl amino acid hydroxamates	2nd Order (M^−1^ s^−1^) 931800	Cat B: 59 Cat S: 10 Papain: 184	Irreversible (In vitro)	Bromme et al., 1993 [110]
8	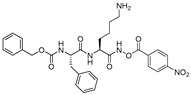	N-peptidyl-O-acyl hydroxamates	2nd Order (M^−1^ s^−1^) 3538000	Cat S: 7 Cat B: 101 Cat H: 4655 SP ^a^: II	Irreversible (In vitro)	Bromme et al., 1993 [109]
9	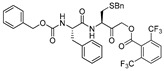	Peptidyl (acyloxy)methanes	2nd Order (M^−1^ s^−1^) 10700000	Cat S: 7 Cat B: 4	Irreversible (In vitro)	Krantz, 1994 [112]
10	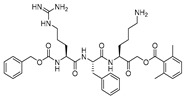	Peptidyl acyloxymethyl ketone	2nd Order (M^−1^ s^−1^) 875000	Cat B: 11	Irreversible (In vitro)	Torkar et al., 2013 [113]
11	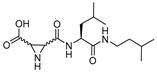	Peptidyl aziridine	2nd Order (M^−1^ s^−1^) (L) ~ 20000 (D) ~ 110000	Cat B ~ 2(L) Cat B ~ 2(D)	Irreversible (In vitro)	Martichonok et al., 1995 [114]
12	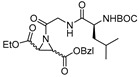	N-acylated aziridines (type I)	2nd Order (M^−1^ min^−1^) (S,S+R,R): 3227	Cat B: 13 Papain: 22	Irreversible (In vitro)	Schirmeister, 1999 [115]
13	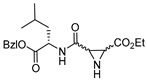	N-unsubstituted aziridines (type II)	2nd Order (M^−1^ min^−1^) (R,R): 16261 (S,S): 3130	Cat B: 10(R,R) Cat B: 76(S,S)	Irreversible (In vitro)	Schirmeister, 1999 [115]
14	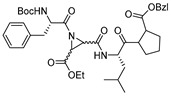	N-acylated bispeptidyl aziridines (type III)	2nd Order (M^−1^ min^−1^) (R,R): 5896 (S,S): 1210	Cat B: 3(R,R) Cat B: 3(S,S)	Irreversible (In vitro)	Schirmeister, 1999 [115]
15	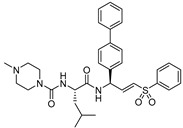	Peptidyl aryl vinyl sulfones	IC_50_ (nM) 2.6	Cat B: 404	Irreversible (In vitro)	Mendieta et al., 2010 [116]
16	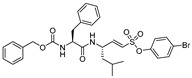	Peptidyl arylvinylsulfonate	2nd Order (M^−1^ s^−1^) 4300000	Cat K: 100 Cat B: 44000 Cat S: 13 Cat H: NI Cat D: NI Cat G: NI hPTP1B: NI Trypsin: NI	Irreversible (In vitro cellular)	Dana et al., 2014 [117]
17	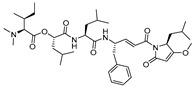	Gallinamide A-analog	2nd Order (M^−1^ s^−1^) 8730000	ND	Irreversible (In vitro cellular)	Boudreau et al., 2019 [118]
18	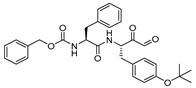	Peptidyl aldehydes	IC_50_ (nM) 0.6	Cat B: 357	Covalent and reversible (In vitro)	Lynas et al., 2000 [119]
19	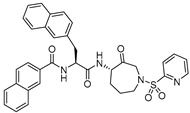	Azepanone-based Inhibitors	K_i_,*_app_* (nM) 0.43	Cat K>20000 Cat S: 36 Cat B: 349	Covalent and reversible (In vitro)	Marquis et al., 2005 [120]
20	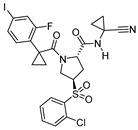	Nitrile group containing inhibitor	IC_50_ (nM) 22	NA	Covalent and reversible (In vitro)	Hardegger et al., 2011 [121]
21	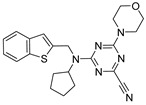	Nitrile group containing inhibitor	Ki (nM) 4	ND	Covalent and reversible (In vitro)	Giroud et al., 2017 [122]
22	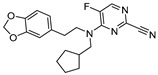	Nitrile group containing inhibitor	K_i_ (nM) 12	ND	Covalent and reversible (In vitro)	Kuhn et al., 2017 [123]
23	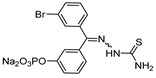	Thiosemicarbazone	IC_50_ (nM) 189 (active inhibitor) ^b^	Cat B: >50	Covalent and reversible (In vitro cellular)	Parker et al., 2017 [124], Parker et al., 2015 [125] Kumar et al., 2010 [126] Kumar et al., 2010 [127]
24	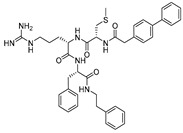	Cat L propeptide mimic	IC_50_ (nM) 19	Cat K: 310 Cat B: 210	Reversible (In vitro)	Chowdhury et al., 2002 [95] Shenoy et al., 2009 [128]
25	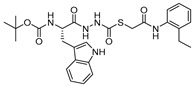	Thiocarbazate	IC_50_ (nM) * 1	Cat V: 11 Cat S: 14 Cat B: 50 Cat K: 137	Reversible (In vitro)	Myers et al., 2008 [129,130,131]
26	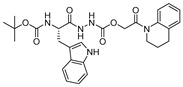	Oxocarbazate	IC_50_ (nM) * 0.4	Cat B: 714	Reversible (In vitro cellular)	Myers et al., 2008 [130] Shah et al., 2010 [132]
27	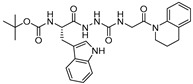	Aza-peptide	IC_50_ (µM) 3.0	ND	Reversible (In vitro)	Myers et al., 2008 [130]

^#^ Fractions were not considered while calculating selectivity factor; Inhibitory efficacy has been reported in whole numbers only; ^a^: Serine Proteases; II: Insignificant Inhibition; ND: Not Determined; ^b^ Active form of inhibitor is devoid of the phosphate group; * Preincubation for 4 h.

**Table 3:** Reported probes of cathepsin L, their detection module, efficacy, and utilities.

#	Chemical Structure	Probe Class	Efficacy (Cathepsin L)	Selectivity Factor	Mechanism of Probe Action (Demonstrated Utilities)	References
P1	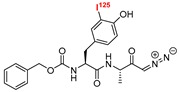	Radio-labelled	2nd order rate constant (M^−1^s^−1^) 240000	Cat B: 23	Covalent and irreversible (In vitro Cellular)	Mason et al., 1989 [165] Wilcox et al., 1992 [166]
P2	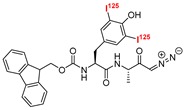	Radio-labelled	2nd order rate constant (M^−1^s^−1^) 60900	Cat B: 2.4 Cat S: NI ^#^	Covalent and irreversible (In vitro Cellular)	Xing et al., 1998 [167]
P3	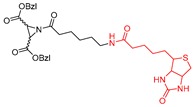	Affinity-labeled	Inhibitor Constant(µM) (S,S) 1.4	Cat B: 36 Papain: 4	Covalent and irreversible (In vitro)	Gelhaus et al., 2004 [168] Vicik et al., 2006 [169]
P4	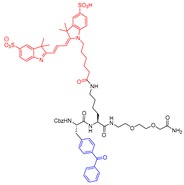	Photoaffinity-based	Inhibitor Constant (µM) 3.6 (able to detect picomolar amount of protein)	Cat B: 9 Cat K: 3 Cat S: 0.3 Cat V: 0.15 [P4 did show remarkable selectivity in vivo and not in vitro]	Covalent and irreversible (In vitro)	Torkar et al., 2012 [170]
5	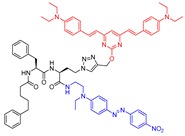	Two-photon FRET-based	N.D	N.D	Reversible (In vitro Cellular)	Na et al., 2012 [171]
6	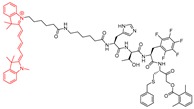	Fluorescent	Cathepsin L specific (some degree of labelling was seen for cat V and B)	Cat V: 847 Cat B: 413 Cat S: 1431 Cat K: >1600	Covalent and irreversible (In vitro Cellular)	Poreba et al., 2018 [172]
7	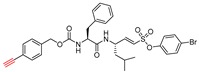	Clickable and tagless	2nd Order (M^−1^ s^−1^) 430000	Cat B:9556 Cat K: 29 Cat H: NI Cat D: NI Cat G: NI hPTP1B: NI Trypsin: NI	Covalent and irreversible (In vitro Cellular)	Dana et al., 2019 [173]
8	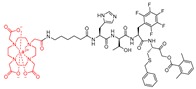	TOF-based	2nd Order (M^−1^ s^−1^) 229300 (mix-Gd) 232500 (159-Tb) 227000 (175-Lu)	Selectivity > 150-fold: cat B, V, and S N.I: cat K and Legumain	Covalent and irreversible (In vitro Cellular)	Poreba et al., 2019 [174]

The detecting agents—radionuclide (P1, P2), biotin (P3), fluorophore (P4, P5, P6), clickable acetylene (P7), and lanthanide containing DOTA (P8)—is red-color coded and the photoactivatable benzoyl group (P4) and quencher (P5) is coded in blue. ^#^: No Inhibition.

**Table 4:** Reported structural studies of cathepsin L enzyme.

PDB Entry	Method	Resolution (Å)	Reference
1CJL	X-ray	2.20	[42]
1CS8	X-ray	1.80	[42]
1ICF	X-ray	2.00	[42]
1MHW	X-ray	1.90	[95]
2NQD	X-ray	1.75	[185]
2VHS	X-ray	1.50	[186]
2XU1	X-ray	1.45	[121]
2XU3	X-ray	0.90	[121]
2XU4	X-ray	1.12	[121]
2XU5	X-ray	1.60	[121]
2YJ2	X-ray	1.15	[97]
2YJ8	X-ray	1.30	[97]
2YJ9	X-ray	1.35	[97]
2YJB	X-ray	1.40	[97]
2YJC	X-ray	1.14	[97]
3BC3	X-ray	2.20	[187]
3H89	X-ray	2.50	[128]
3H8B	X-ray	1.80	[128]
3H8C	X-ray	2.50	[128]
3HHA	X-ray	1.27	[147]
3HWN	X-ray	2.33	[147]
3IV2	X-ray	2.20	[188]
3K24	X-ray	2.50	[188]
3KSE	X-ray	1.71	[189]
3OF8	X-ray	2.20	[96]
3OF9	X-ray	1.76	[96]
4AXL	X-ray	1.92	[190]
4AXM	X-ray	2.80	[190]
5F02	X-ray	1.43	[191]
5I4H	X-ray	1.42	[192]
5MAE	X-ray	1.00	[145]
5MAJ	X-ray	1.00	[145]
5MQY	X-ray	1.13	[123]
6EZP	X-ray	1.37	[98]
6EZX	X-ray	2.34	[98]
6F06	X-ray	2.02	[98]

## Abbreviations

MA	Methacrylate
Phe	Phenylalanine
Gly	Glycine
Z/Cbz	Carboxybenzyl
His	Histidine
NBz	Nitrobenzyl
iAm	isoamyl
SAR	Structure Activity Relationship
AOMK	Acyloxymethanes/Acyloxymethyl Ketones
Leu	Leucine
Met	Methionine
Azi	Aziridine
FRET	Fluorescence Resonance Energy Transfer
catABP	Clickable and Tagless Activity-based Probe
